# A modified white shark optimizer for optimal power flow considering uncertainty of renewable energy sources

**DOI:** 10.1038/s41598-024-53249-z

**Published:** 2024-02-06

**Authors:** Mohamed Farhat, Salah Kamel, Mohamed A. Elseify, Almoataz Y. Abdelaziz

**Affiliations:** 1 Electrical Power and Machines Engineering Department, Faculty of Engineering, Ain Shams University, Cairo, 11517 Egypt; 2 Department of Electrical Engineering, Faculty of Engineering, Aswan University, Aswan, 81542 Egypt; 3 Department of Electrical Engineering, Faculty of Engineering, Al-Azhar University, Qena, 83513 Egypt; 4 Faculty of Engineering and Technology, Future University in Egypt, Cairo, 11835 Egypt

**Keywords:** Engineering, Electrical and electronic engineering

## Abstract

This paper presents a novel approach to solve the optimal power flow (OPF) problem by utilizing a modified white shark optimization (MWSO) algorithm. The MWSO algorithm incorporates the Gaussian barebones (GB) and quasi-oppositional-based learning (QOBL) strategies to improve the convergence rate and accuracy of the original WSO algorithm. To address the uncertainty associated with renewable energy sources, the IEEE 30 bus system, which consists of 30 buses, 6 thermal generators, and 41 branches, is modified by replacing three thermal generators with two wind generators and one solar PV generator. And the IEEE 57-bus system, which consists of 57 buses, 7 thermal generators, and 80 branches, is also modified by the same concept. The variability of wind and solar generation is described using the Weibull and lognormal distributions, and its impact on the OPF problem is considered by incorporating reserve and penalty costs for overestimation and underestimation of power output. The paper also takes into account the unpredictability of power consumption (load demand) by analyzing its influence using standard probability density functions (PDF). Furthermore, practical conditions related to the thermal generators, such as ramp rate limits are examined. The MWSO algorithm is evaluated and analyzed using 23 standard benchmark functions, and a comparative study is conducted against six well-known techniques using various statistical parameters. The results and statistical analysis demonstrate the superiority and effectiveness of the MWSO algorithm compared to the original WSO algorithm for addressing the OPF problem in the presence of generation and demand uncertainties.

## Introduction

The optimization of power flow was initially developed by Carpentier in 1962^1^. Afterward, several methods have been developed for addressing the optimal power flow (OPF) problem. The OPF is used to minimize power losses, maintain voltage stability, optimize generating costs, and eliminate gas emissions. The physical limits of the power network, which include the need to comply with power generator capability, buses’ voltage, capacities of transmission lines, power cable flows, and any other technical requirements, typically constrain this optimization. This may seem like a complex issue, particularly in high-power systems. Therefore, particular measures should be taken to prevent exceeding these physical boundaries. The classical OPF only consists of fossil fuel-fired conventional generating sources, which creates an exceedingly mixed integer, non-linear, and non-convex optimization issue^2–4^. The increasing inclusion of renewable energy into electrical networks necessitates the inclusion of its uncertain character in OPF studies because of the accompanying issues throughout the operational and planning stages. Several traditional optimization methods have been developed to deal with the OPF challenge. These techniques include quadratic programming, non-linear programming, mixed-integer linear programming, and interior-point techniques^5,6^. Certain strategies have been successfully employed in the industry because of their quick convergence and ability to provide the optimum solution. However, these optimization techniques necessitate first linearizing the optimization function. On the other hand, some heuristic optimization strategies have been proposed as a potential solution to address this issue^7^. For this reason, the OPF is solved using a variety of heuristic approaches.

The OPF problem was addressed using a sequential GA solution approach in combination with a simple genetic algorithm (SGA) to acquire a suitable control variable resolution without violating system constraints^8^. In Ref.^9^, a dependable and effective Tabu search best approach has been proposed and assessed on the IEEE 30-bus power network. Numerous earlier studies have relied on differential evolution to rectify the OPF problem. These studies have quick convergence characteristics and are appropriate for OPF problems with complex variables. Nevertheless, there is a significant chance that they will converge to a local instead of a global optimal solution^10–12^. In several challenging OPF issues, particle swarm optimization has been applied. The premature convergence is a major disadvantage of classical PSO, as it is with many heuristic techniques^13–15^. Grey wolf optimization^16^, artificial bee colony^17^, flower pollination algorithm^18^, cuckoo search optimization^19^, crow search algorithms^20^, success history-based adaptive differential evolution algorithm^21^, group search optimization^22^, JAYA algorithm^23^, moth swarm algorithm^24^, golden ratio optimization method^25^, and Aquila optimizer^26^, barnacle mating optimizer^27^, mayfly algorithm^28^, coronavirus herd immunity algorithm^29^, and weighted mean of vectors (INFO) algorithm^30^ are just a few of the meta-heuristic population-based algorithms that have been employed in the past few years to solve OPF problems.

Various adjustments have been made to metaheuristic optimization approaches in the literature to address the issue of early convergence and provide an improved solution for the OPF problem, such as modified JAYA^31^, enhanced bacteria foraging algorithm (MBFA)^32^, SHADE-SF^33^, modified grasshopper optimization^34^, improved rao-2 algorithm^35^, boosted quasi-reflection jellyfish optimization algorithm^36^, hybrid cross entropy-cuckoo search algorithm^37^, hybrid TLTFWO^38^ through the integration between the teaching and learning algorithm and turbulent flow of water algorithm, hybrid Mayfly algorithm and Aquila optimizer^39^. Accordingly, this study aims to develop a recent optimization technique named white shark optimization (WSO) to tackle the OPF, considering several real-world scenarios and the uncertainties associated with the generation and demand.

Freshly, the WSO algorithm was developed by Malik et al.^40^ in 2022 and applied for handling most complex optimization challenges, such as solving uncertain optimal power flow^41^ and distributed generation optimal allocation^42^. However, WSO has some drawbacks, such as a slow convergence rate and an imbalance between the exploration and exploitation phases. In the literature, some studies have been conducted to overcome such limitations. In Ref.^43^, the authors proposed a method for adjusting the force control parameters of the WSO by including a chaotic generator to enhance the exploitation capabilities of the algorithm. Further, the authors in Ref.^44^ provided a suggested methodology involves adjusting the probability parameters of WSO to align with the optimization process and effectively synchronize all phases of the algorithm’s search process. Furthermore, they incorporated wave theory to elucidate the equation governing the trajectory motion of fluid particles inside the micro amplitude wave theory. The exploration process is also enhanced by incorporating the spiral search technique from the whale optimization algorithm. In Ref.^45^, the authors proposed a new hybrid WSO and whale optimization algorithm to improve the stochastic behavior of the WSO algorithm for specifying the appropriate parameters of Li-ion battery Shepherd model equivalent circuits. Also, WSO is hybridized with the equilibrium optimizer for utilizing IOT for power scheduling problems^46^. In this work, the Gaussian barebones (GB) and quasi-oppositional-based learning (QOBL) strategies are incorporated into the original WSO algorithm to enhance its convergence speed and accuracy while addressing the complicated optimal power flow problem.

The developed MWSO is evaluated via 23 benchmark functions, which include unimodal, high-dimensional multi-modal, and fixed high-dimensional multi-modal functions, and a comparison with other six rivals is conducted using different statistical analysis. These algorithms comprise particle swarm optimization (PSO)^47^, whale optimization algorithm (WOA)^48^, salp swarm algorithm (SSA)^49^, Kepler optimization algorithm (KOA)^50^, nutcracker optimizer algorithm (NOA)^51^, and the traditional WSO. Then, the MWSO algorithm is employed to solve the optimal power flow problem on the modified IEEE 30-bus and 57-bus power networks, considering different real-world scenarios. Eventually, the key effort of this research can be listed as follows:

- Introducing a modified white shark optimization algorithm (MWSO) by incorporating Gaussian barebones and quasi-oppositional-based learning to enhance exploration capabilities and improve convergence rates compared to the original WSO.
- Validating the effectiveness of the MWSO algorithm by applying it to 23 benchmark functions and comparing its performance against efficient competitors using various statistical metrics.
- Modifying the IEEE 30-bus to include wind and solar power plants, and utilizing both the MWSO and original WSO algorithms to address the optimal power flow (OPF) problem through four different objective functions.
- Conducting practical scenarios that consider the uncertainty of generation and demand, as well as ramp rate limits of thermal power plants, and analyzing the results obtained from the proposed MWSO algorithm and the original WSO algorithm in these simulation scenarios.
- Using a modified IEEE 57-bus system to demonstrate the scalability of the proposed MWSO.

The obtained results clearly demonstrate the superiority and dominance of the developed MWSO algorithm over the traditional WSO algorithm in effectively addressing the OPF problem.

The outstanding portions of the present study are: “Different cost models” section outlines the different cost models that include thermal, wind, and solar power costs. “Objective functions and system constraints” section presents the various OPF objective functions and corresponding constraints. Then, “Proposed MWSO methodology” section presents the modified algorithm (MWSO). The simulation results, comprising real-world case studies using the MWSO and WSO methods, are given in “Simulation results and discussion” section, in addition to the statistical analysis using the Wilcoxon signed rank test. Also, this section includes the experimental results and discussions of the 23 benchmark testing functions. Finally, “Conclusion” section concludes the findings and future recommendations of the paper.

## Different cost models

In this study, some modifications are applied on the IEEE 30-bus test network to include wind and solar plants. At buses 5 and 11, the two thermal plants have been replaced by two wind power plants, and the thermal plant at bus 13 has also been replaced by a solar PV plant^33^. The IEEE 57-bus system is also reformed by changing the thermal plants at buses 2 and 6 with two wind plants and changing the thermal plant at bus 9 with solar PV plant^52^. The data of the wind and solar plants of the IEEE 30-bus system and the IEEE 57-bus system are provided in Supplementary Material Tables 1A and 4A, respectively. This section will provide a detailed explanation of the production costs of each power source in the IEEE 30-bus power system. Since the production costs of the IEEE 57-bus power system follow the same procedure, they will not be explained here.

### Cost of thermal power

The produced thermal power charges a cost that can be calculated using (1), where the valve point impact of thermal plants has been taken into consideration while calculating the cost of thermal power to provide more accurate values.

1 $${C}_{Th}({P}_{Th})=\sum \limits_{i=1}^{{N}_{Th}}{a}_{Thi}+{b}_{Thi}{P}_{Thi}+{c}_{Thi}{{P}^{2}}_{Thi} +\left|{d}_{Thi}\times {\text{sin}}({e}_{Thi}\times ({P}_{Thi}^{min}-{P}_{Thi}))\right|,$$

where ${P}_{Thi}$ is the output power of the $i\text{-th}$ thermal plant, while ${a}_{Thi}$, ${b}_{Thi}$, and ${c}_{Thi}$ indicate the cost coefficients of the $i\text{-th}$ thermal plant. ${N}_{Th}$ indicates the number of thermal plants, while ${d}_{Thi}$ and ${e}_{Thi}$ indicate the coefficients of valve point loading, and ${P}_{Thi}^{min}$ denotes the minimum amount of power produced from the $i\text{-th}$ thermal plant. The values of all mentioned coefficients in this equation are listed in Supplementary Material Table 2A.

### Components of wind power cost

In contrast to thermal power, wind power is subject to considerable uncertainty. Accordingly, the cost of production using wind is computed differently, as stated below.

#### Direct component

The power that is intended to be generated by wind turbines has a direct cost that can be estimated as follows:

2 $${C}_{directwj}={dw}_{j}{P}_{schwj},$$

where ${P}_{schwj}$ represents the intended wind power of the $j\text{-th}$ wind plant and ${dw}_{j}$ indicates the coefficient of its direct charge.

#### Uncertain components

Given the variable character of wind power, two scenarios are possible. The first of these scenarios comes about if the actual production of wind turbines is less than what was anticipated to be produced. This is known as overestimation, and a commitment to the spinning reserve must be made to compensate for it. According to that, a reserve cost is required, which is computed as follows:

3 $${C}_{reserve\, wj}={K}_{reswj}\left({P}_{schwj}-{P}_{available\, wj}\right)={K}_{reswj}\int \limits_{0}^{{P}_{sch wj}}\left( {P}_{schwj}-{ P}_{wind\, j}\right){f}_{wind\, j}\left({P}_{wind\, j}\right)d{P}_{wind\, j},$$

where ${K}_{reswj}$ corresponds to the reserve cost coefficient for the $j\text{-th}$ wind power plant and ${P}_{available\, wj}$ signifies the actual available power from the same plant. The PDF of the wind power from the $j\text{-th}$ wind plant is signed as ${f}_{wind\, j}$.

In the second scenario, the amount of electrical power actually provided by the wind turbines may be greater than what was anticipated. If it is not possible to use the extra electrical power, traditional generators’ output must be reduced. A penalty fee equal to the excessive power is due from ISO. The definition of the penalty cost corresponding to a wind plant can be clarified by (4):

4 $${C}_{penalty\, wj}={K}_{pen\, wj}({P}_{available\, wj}-{P}_{schwj})= {K}_{pen\, wj}\int \limits_{{P}_{schwj}}^{{P}_{rated \,wj}}({P}_{wind\, j}-{P}_{schwj}){f}_{wind\, j}({P}_{wind\, j})d{P}_{wind\, j},$$

where, ${K}_{penwj}$ signifies the penalty cost coefficient, and ${P}_{rated \,wj}$ states to the rated power of a wind plant $(j)$.

### Probabilistic power of wind plants

In this part, the probabilistic power of wind plants, the term “${f}_{wind\, j}({P}_{wind\, j})$” in (3) and (4), will be determined. The Weibull probability density function (PDF) works well with the wind speed distribution^32,53^. Following the Weibull PDF, the following formula is utilized for calculating the probability of wind speed $({Wind}_{v})$:

5 $${f}_{{Wd}_{v}}\left({Wd}_{v}\right)=\left(\frac{k}{c}\right)+{\left(\frac{{Wd}_{v}}{c}\right)}^{\left(k-1\right)}{e}^{{-\left({{Wd}_{v}}/{c}\right)}^{k}}\,for\, 0<{Wd}_{v}<\infty ,$$

where the letters $k$ and $c$, respectively, stand for scale and form factors. Weibull distribution’s mean is calculated as follows:

6 $${M}_{weibull}=c\times\Gamma \left(1+{k}^{-1}\right).$$

The gamma function, which is represented by the sign $\Gamma$ in (6), is provided by:

7 $$\Gamma \left(x\right)=\int \limits_{0}^{\infty }{e}^{-t}{t}^{x-1}dt.$$

**Figure 1:**
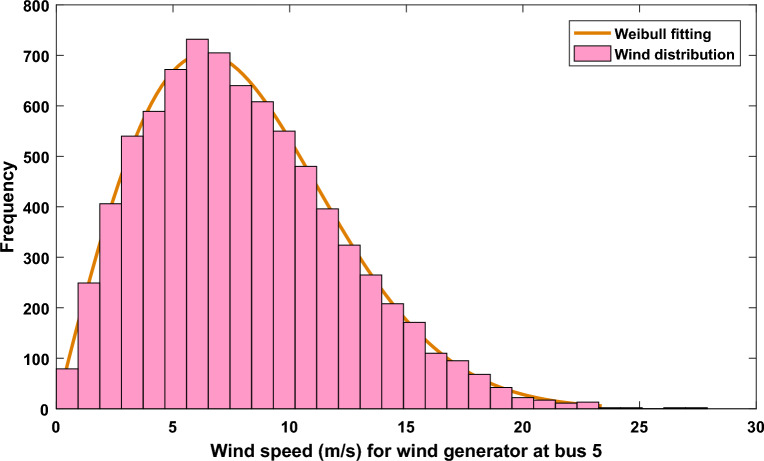
Wind speed distribution and Weibull fitting for wind plant at bus 5.

**Figure 2:**
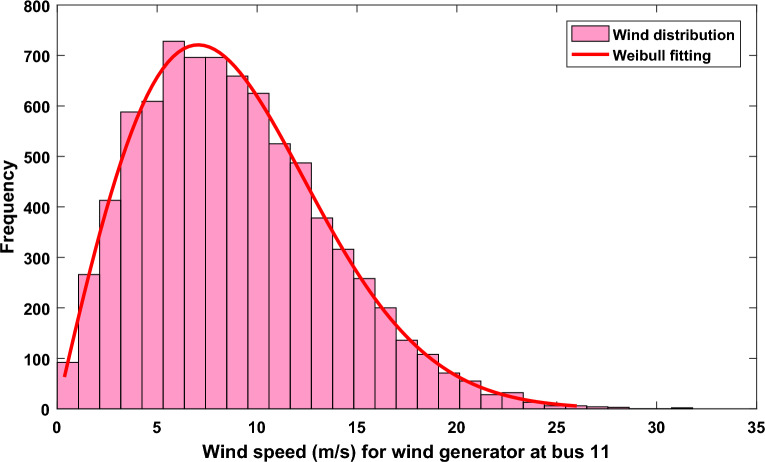
Wind speed distribution and Weibull fitting for wind plant at bus 11.

After conducting 8000 Monte–Carlo simulation scenarios, Figs. 1 and 2 reveal the frequency distribution of the wind based on Weibull fitting for the wind plant at bus 5 and the wind plant at bus 11, respectively. The applied values of the Weibull distribution have been listed in Supplementary Materials Table 1A.

The wind speed influences the wind plant’s output power of. According to Ref.^33^, the following is the formula for wind turbine power output:

8 $${P}_{wind}\left({Wd}_{v}\right)=\left\{\begin{array}{*{20}l}0\, for\, {Wd}_{v}<{Wd}_{vin} \,and \,{Wd}_{v}>{Wd}_{vout} & \quad (discrete \,region )\\ {P}_{rated w}\times \left(\frac{({Wd}_{v}-{Wd}_{vin}}{({Wd}_{vr}-{Wd}_{vin}}\right)\,for\, {Wd}_{vin}\le {Wd}_{v}\le {Wd}_{vr} & \quad \left(continous\, region \right)\\ {P}_{rated\, w} \,for\, {Wd}_{vr}\le {Wd}_{v}\le {Wd}_{vout} &\quad \left(discrete \,region \right)\end{array}\right..$$

In this formula, the cut-in speed is shown by ${Wd}_{vin}$, the cut-out speed is indicated by ${Wd}_{vout}$, and the rated wind speed is indicated by ${Wd}_{vr}$. The wind turbine’s rated power is shown by the variable ${P}_{rated w}$.

It is possible to establish the probability of output power from wind plant in the discrete region as follows^54^:

9 $${f}_{wind}\left({P}_{wind}\right)\left\{{P}_{wind}=0\right\}=1-{\text{exp}}\left[-{\left(\frac{{Wd}_{vin}}{c}\right)}^{k}\right]+{\text{exp}}\left[-{\left(\frac{{Wd}_{vout}}{c}\right)}^{k}\right],$$

10 $${f}_{wind}\left({P}_{wind}\right)\left\{{P}_{wind}={P}_{rated w}\right\}={\text{exp}}\left[-{\left(\frac{{Wd}_{vr}}{c}\right)}^{k}\right]-{\text{exp}}\left[-{\left(\frac{{Wd}_{vout}}{c}\right)}^{k}\right].$$

Regarding the continuous zone, the following formula can be used to determine the probabilities for the power that the wind plant will produce:

11 $${f}_{wind}\left({P}_{wind}\right)=\frac{k\left({Wd}_{vr}-{Wd}_{vin}\right)}{{c}^{k}\times {P}_{rated w}}{\left[{Wd}_{vin}+\frac{{P}_{wind}}{{P}_{rated w}}\left({Wd}_{vr}-{Wd}_{vin}\right)\right]}^{k-1}\times {\text{exp}}\left[{-\left(\frac{{Wd}_{vin}+\frac{{P}_{wind}}{{P}_{rated w}}\left({Wd}_{vr}-{Wd}_{vin}\right)}{c}\right)}^{k}\right].$$

### Components of solar power cost

The total cost of producing electricity from solar system may be broken down into a direct cost and an uncertainty cost, much like the cost of wind power.

#### Direct component

The direct component of solar power cost is estimated using (12).

12 $${C}_{directsk}={ds}_{k}{P}_{schsk},$$

where ${P}_{schsk}$ represents the intended solar power of the $k\text{-th}$ wind plant and ${ds}_{k}$ indicates the coefficient of its direct cost.

#### Uncertain components

Similar to how wind energy is estimated, the cost of producing power from solar plants is determined in both overestimation and underestimation scenarios. Consequently, the reserve cost of solar power in case of overestimating is determined by the following formula:

13 $${C}_{reserve\, sk}={K}_{ressk}\left({P}_{schsk}-{P}_{available \,sk}\right)= {K}_{ressk}\times {f}_{solar k}\left({P}_{available \,sk}<{P}_{sch sk}\right)\times \left[({P}_{schsk}-E({P}_{available \,sk}<{P}_{schsk})\right].$$

As, ${K}_{ressk}$ indicates the reserve charge coefficient for the solar plant $(k)$, and ${P}_{available \,sk}$ signifies the available output of the same solar plant, while ${f}_{solar k}({P}_{available \,sk}<{P}_{schsk})$ signifies the deficiency existence probability in the production of solar plant, and the expectation of being the output of solar plant below the ${P}_{schsk}$ is denoted by $E({P}_{available \,sk}<{P}_{schsk})$. And the penalty cost of solar power in case of underestimating is determined by:

14 $${C}_{penalty sk}={K}_{pen sk}\left({P}_{available \,sk}-{P}_{schsk}\right)= {K}_{pensk}\times {f}_{solar k}\left({P}_{available \,sk}>{P}_{schsk}\right)\times \left[(E({P}_{available \,sk}<{P}_{sch sk})-{P}_{schsk}\right],$$

where, ${K}_{pensk}$ signifies the penalty cost coefficient, ${f}_{solar k}({P}_{available \,sk}>{P}_{schsk})$ expresses the probability of the unused solar power produced from the solar plant $(k)$, while $E({P}_{available \,sk}<{P}_{schsk})$ signifies the expected remaining output power from the solar plant $(k)$.

### Probabilistic power of solar plant

The variable solar irradiance $(I)$ impacts the output power of the solar plant. Equation (15) provides a clarification on how the probability of sun irradiance is calculated based on the lognormal PDF^55^.

15 $${f}_{I}\left(I\right)=\frac{1}{I\sigma \sqrt{2\pi }}{\text{exp}}\left\{\frac{-{\left({\text{ln}}x-\mu \right)}^{2}}{{2\sigma }^{2}}\right\}for I>0.$$

As, the irradiance probability is denoted by ${f}_{I}(I)$, the mean of the lognormal distribution is denoted by $\mu$, while the standard deviation is signified by $\sigma$, respectively. While the mean of lognormal, ${M}_{lgn}$ is calculated by:

16 $${M}_{lgn}={\text{exp}}\left(\mu +\frac{{\sigma }^{2}}{2}\right).$$

**Figure 3:**
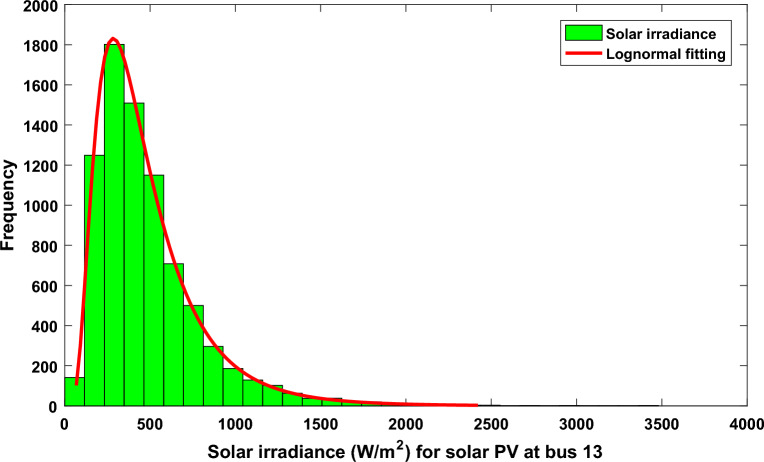
Distribution of irradiance and lognormal fitting for solar PV at bus 13.

In this regard and after performing 8000 Monte–Carlo scenarios, the lognormal distribution for the solar plant is shown in Fig. 3. The applied values of the lognormal distribution are listed in Supplementary Material Table 1A.

Consequently, the sun irradiation vs. the energy conversion of the solar plant can be presented as follows^56^:

17 $${P}_{solar}\left(I\right)=\left\{\begin{array}{l}{P}_{solarr}\left(\frac{{I}^{2}}{{I}_{std}{R}_{c}}\right)\, for\, 0<I<{R}_{c}\\ {P}_{solarr}\left(\frac{I}{{I}_{std}}\right)\, for\, I\ge {R}_{c}\end{array}\right.,$$

where in this formula, ${I}_{std}$ signifies the solar irradiance when the environment is a standard i.e. (800 W/m^2^), the symbol ${R}_{c}$ specifies a specific value of irradiance (120 W/m^2^), and ${P}_{solarr}$ refers to the rated power of the solar PV system.

**Figure 4:**
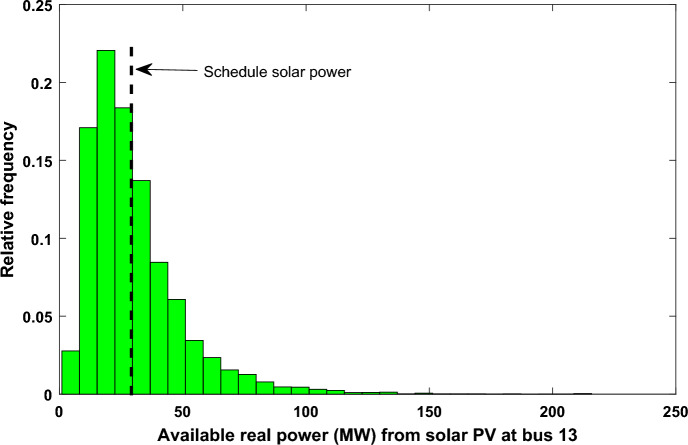
Available real solar PV power at bus 13.

The reserve charge of the solar power that are stated in (13) can be rewritten after determining the probabilities of solar power as follows:

18 $${C}_{reserve\, sk}={K}_{ressk}\left({P}_{schsk}-{P}_{available \,sk}\right)= {K}_{ressk}\times \sum_{n=1}^{{N}^{-}}\left[{P}_{schsk}-{P}_{sn-}\right]\times {f}_{sn-},$$

where ${P}_{sn-}$ signifies the unavailability of solar power (lesser than the schedule power) as indicated by the left half plane of the schedule power of the solar plant (${P}_{schsk}$) inside Fig. 4. ${f}_{sln-}$ signifies the relative frequencies of the ${P}_{sn-}$ occurrence. ${N}^{-}$ signifies the number of discrete bins on the left plane of the schedule power of the solar plant.

While, the penalty cost that are previously stated in (14) can be rewritten as follows:

19 $${C}_{penalty sk}={K}_{pensk}\left({P}_{available \,sk}-{P}_{schsk}\right)={K}_{pensk}\sum_{n=1}^{{N}^{+}}\left[{P}_{sn+}-{P}_{schsk}\right]\times {f}_{sn+},$$

where, ${P}_{sn+}$ signifies the surplus of solar power (higher than the schedule power) as indicated by the right half plane of the schedule power of the solar plant (${P}_{sch sk}$) provided in Fig. 4. ${f}_{sln+}$ gives the relative frequencies of the ${P}_{sn+}$ occurrence. ${N}^{+}$ signifies the number of discrete bins on the right plane of the schedule power of the solar plant.

The reserve charge coefficient (${K}_{res}$) for the two wind plants and the solar plant are constant value equals to 3 for all of them, and the penalty charge coefficient (${K}_{pen}$) equals to 1.5 for all of them. The direct charge coefficient of the two wind plants ${(dw}_{j})$ and the solar plants ${(ds}_{k})$ equals to 1.75, while the direct charge coefficient for the solar plant equals to 1.6. Unless otherwise specified, these values will be utilised in the case studies in “Conclusion” section.

### Cost of carbon emissions

The thermal plants are sources for carbon emissions (${\text{CAE}})$, thus Eq. (20) provides an estimation for these emissions.

20 $$CAE=\left(\sum \limits_{i=1}^{{N}_{Th}}\left({\varphi }_{Th,i}+{\Psi }_{Th,i}\;{P}_{Thi}+{\omega }_{Th,i}\;{{P}^{2}}_{Thi}\right)+{\tau }_{Th,i}\;{e}^{({\zeta }_{Th,i}\;{P}_{Thi})}\right).$$

Here, $${\varphi }_{Th,i}$$, $${\Psi }_{Thi}$$, $${\omega }_{Th,i}$$, $${\tau }_{Th,i}$$, and $${\zeta }_{Th,i}$$ signify emissions coefficients of the thermal plants. The values of these coefficients are given in Supplementary Materials Tables 2A and 3A for the two systems. These emissions in tonnes are translated into cost, $${C}_{CE}$$ in $/h as per Eq. (21), where, $${C}_{Taxc}$$ is the tax of emissions in $/tonne.

21 $${C}_{CE}=CAE\times {C}_{Taxc}.$$

## Objective functions and system constraints

### Objective functions

Minimizing the total cost of production with and without enforcing a tax on carbon emissions, minimizing carbon emissions, and minimizing power losses are the objective functions of this optimal power flow model. The different objective functions of this model can be expressed as follows.

#### Minimizing the total cost of production without enforcing tax on carbon emissions $({F}_{1})$

${F}_{1}$ Can be formulated by combining all the above-mentioned different costs in “Different cost models” section. Therefore, ${F}_{1}$ can be written as:

22 $${F}_{1} =\text{ min}\left({C}_{Th}\left({P}_{Th}\right)+\sum \limits_{j=1}^{{N}_{WP}}\left({dw}_{j}{P}_{schwj}+{K}_{reswj}\left({P}_{schwj}-{P}_{available\, wj}\right)+{K}_{penwj}({P}_{available\, wj}-{P}_{schwj})\right)+\sum \limits_{j=1}^{{N}_{SP}}\left({ds}_{k}{P}_{schsk}+{K}_{ressk}\left({P}_{schsk}-{P}_{available \,sk}\right)+{K}_{pensk}({P}_{available \,sk}-{P}_{schsk})\right)\right).$$

#### Minimizing the total cost of production with enforcing tax on carbon emissions $({F}_{2})$

${F}_{2}$ Can be formulated by adding all the costs in ${F}_{1}$ into the emissions cost that was expressed in (21). Therefore, ${F}_{2}$ can be written as:

23 $${F}_{2}=min\left({C}_{Th}\left({P}_{Th}\right)+\sum \limits_{j=1}^{{N}_{WP}}\left[{dw}_{j}{P}_{schwj}+{K}_{reswj}\left({P}_{schwj}-{P}_{available\, wj}\right)+{K}_{penwj}\left({P}_{available\, wj}-{P}_{schwj}\right)\right]+\sum \limits_{j=1}^{{N}_{SP}}\left[{ds}_{k}{P}_{schsk}+{K}_{ressk}\left({P}_{schsk}-{P}_{available \,sk}\right)+{K}_{pensk}\left({P}_{available \,sk}-{P}_{schsk}\right)\right]+\left[CAE\times {C}_{Tax}\right]\right).$$

#### Minimizing the carbon emissions $({F}_{3})$

${F}_{3}$ Is to minimize the total carbon emissions of the thermal plants in (20). Therefore, it can be written as:

24 $${F}_{3}=min\left(\sum \limits_{i=1}^{{N}_{Th}}\left[{\varphi }_{Th,i}+{\Psi }_{Th,i}\;{P}_{Thi}+{\omega }_{Th,i}\;{{P}^{2}}_{Thi}+{\tau }_{Th,i}\;{e}^{({\zeta }_{Th,i}\;{P}_{Thi})}\right]\right).$$

#### Minimizing the total power losses $({F}_{4})$

The power losses of the power system can be formulated as follows:

25 $${P}_{loss}=\left(\sum_{i=1}^{NTL}\sum_{j\ne 1}^{NTL}\left[{G}_{ij}{V}_{i}^{2}+{V}_{j}^{2}-2{V}_{i} {V}_{j}{\text{cos}}({\delta }_{ij})\right]\right),$$

${\text{where }\delta }_{ij}$ expresses the difference in voltage angles between buses $i$ and $j$; $NTL$ signifies the number of transmission lines; and ${G}_{ij}$ expresses the transfer conductance. Consequently, ${F}_{4}$ can be formulated as follows:

26 $${F}_{4}=min\left(\sum_{i=1}^{NTL}\sum_{j\ne 1}^{NTL}\left[{G}_{ij}{V}_{i}^{2}+{V}_{j}^{2}-2{V}_{i} {V}_{j}{\text{cos}}({\delta }_{ij})\right]\right).$$

### Problem constraints

The problem of OPF is constrained by some conditions that must not be violated. Some of these constraints are equality and the others are inequality constraints.

#### Equality constraints

The equality constraints are dedicated to ensuring that the generated (active and reactive) power in the system equals to the consumed (actives and reactive) power in addition to the power loss:

27 $$P_{Gi} = P_{Di} + V_{i} \mathop \sum \limits_{j = 1}^{NB} V_{j} \left[ {G_{ij} {\text{cos}}\left( {\delta_{ij} } \right) + B_{ij} {\text{sin}}\left( {\delta_{ij} } \right)} \right]{ }\quad \forall i \in NB{ },$$

28 $$Q_{Gi} = Q_{Di} + V_{i} \mathop \sum \limits_{j = 1}^{NB} V_{j} \left[ {G_{ij} {\text{sin}}\left( {\delta_{ij} } \right) - B_{ij} {\text{cos}}\left( {\delta_{ij} } \right)} \right]\quad \forall i \in NB,$$

where $NB$ stands for the network buses number. ${{B}_{ij}\text{ and }G}_{ij}$ stand for the susceptance and conductance among bus $i$ and bus $j$, respectively. ${\delta }_{ij}$ is the voltage angle of bus i minus the voltage angle of bus $j$. The real components of the produced and consumed power at bus $i$ are expressed by ${P}_{Gi}\, {\text{and}}\, {P}_{Di}$, respectively, while the reactive components of the consumed and generated power are expressed by ${Q}_{Di}{ \,{\text{and}}\, Q}_{Gi}$, respectively.

#### Inequality constraints

These constraints define upper and lower limits for the operation of system components such as the generators, transmission lines, and load buses.

##### Generator limits

Lower and higher limits govern the functioning of all power generators in the network. There are limits for the active power, reactive power, and voltage of the generator as showed by (29), (30), and (31), respectively, where ${N}_{G}$ represents the number of network’s generators.

29 $${P}_{Gi}^{min}\le {P}_{Gi}\le {P}_{Gi}^{max}, i=1,\ldots ,{N}_{G},$$

30 $${Q}_{Gi}^{min}\le {Q}_{Gi}\le {Q}_{Gi}^{max}, i=1,\ldots, {N}_{G},$$

31 $${V}_{Gi}^{min}\le {V}_{Gi}\le {V}_{Gi}^{max}, i=1,\ldots ,{N}_{G}.$$

##### Transformer limits

32 $${T}_{t}^{min}\le {T}_{t}\le {T}_{t}^{max}, t=1,\ldots ,{N}_{T}.$$

##### Shunt compensator limits

33 $${Q}_{Cc}^{min}\le {Q}_{Cc}\le {Q}_{Cc}^{max}, c=1,\ldots ,{N}_{C},$$

where N_T_ and N_C_ refer to the number of transformers and shunt compensators in the network, respectively.

##### Limits of ramp rate

The ramp-rate restrictions of thermal generators can be identified in the following manner:

34 $${{{\text{P}}}_{{\text{Thi}}}-{\text{P}}}_{{\text{Thi}}}^{0}\le {{\text{Ur}}}_{{\text{i}}},\text{ if power generation rises },$$

35 $${{\text{P}}}_{{\text{Thi}}}^{0}-{{\text{P}}}_{{\text{Thi}}}\le {{\text{Dr}}}_{{\text{i}}},\text{ if power generation reduces},$$

where,${P}_{Thi}^{0}$ signifies the previous hour’s output power from the thermal plant $( i )$. ${Ur}_{i} {\text{and}} {Dr}_{i}$ signify the up and down limits of ramp-rate for the i-th thermal plant, respectively.

##### Limits of load buses

The voltages of load buses are constrained by lower and upper limits that can be clarified as follows:

36 $${{\text{V}}}_{{{\text{LB}}}_{{\text{p}}}}^{{\text{min}}}\le {{\text{V}}}_{{{\text{LB}}}_{{\text{p}}}}\le {{\text{V}}}_{{{\text{LB}}}_{{\text{p}}}}^{{\text{max}}},\text{ p}=1,\dots .\dots \dots ,{{\text{N}}}_{{\text{LB}}},$$

where ${N}_{LB}$ denotes the load buses number in the grid. There is another important parameter related to the load buses, which is the voltage deviation of load buses which can be calculated as follows:

37 $${V}_{d}=\sum_{p=1}^{{{\text{N}}}_{{\text{LB}}}}\left|{V}_{LBp}-1\right|.$$

It is a measure of the quality system’s voltage. This indicator is defined as the total deviation of all load buses buses in the system from the nominal value of 1 p.u.

##### Capacity of transmission lines

The capacity of the transmission lines in the network should not exceed a certain limit. This constraint can be clarified as per Eq. (38), where ${N}_{L}$ denotes the of transmission lines number in the grid.

38 $${S}_{{L}_{q}}\le {S}_{{L}_{q}}^{max} , q=1,\ldots ,{N}_{L}.$$

## Proposed MWSO methodology

### Original WSO algorithm: an overview

This section clarifies a short description of the mathematical formulation of the original WSO^40^, which was designed to depict how white sharks behave when foraging. This involves pursuing and chasing prey. The great white shark is capable of locating prey (i.e., a food source) at the depths of the ocean. The location of the food supply in a specific search area is unknown, though. In this situation, white sharks must conduct extended searches to find food sources in the ocean’s depths. The three activities of the great white sharks to identify prey (i.e., the best food source) are as follows: (1) moving towards prey is based on the waves’ hesitancy as a result of their movement. In this situation, the white shark utilizes a wavy movement to locate prey using its related sense of hearing and smell. As well as in (2) its haphazard quest for prey in the ocean’s depths. Great white sharks do this by swimming in the direction of their prey and remaining close to the best one, and (3) white shark behavior in seeking the nearest prey. In doing so, the great white shark mimics fish school behavior by swimming toward the best white shark that is close to the optimum prey. Based on such actions, all white shark sites are adjusted around the global possible solutions if the prey is not discovered properly. The mathematical expressions for these activities are as follows.

#### Initialization

The WSO algorithm is a population-based algorithm like several metaheuristic optimization techniques. The candidate solutions to an optimization problem with an $n$ population size (i.e., white shark) and $d$ dimensional space are expressed as per Eq. (39).

39 $$w=\left[\begin{array}{ccc}\begin{array}{cc}\begin{array}{l}{w}_{1}^{1}\\ {w}_{1}^{2}\end{array}& \begin{array}{l}{w}_{2}^{1}\\ {w}_{2}^{2}\end{array}\end{array}& \begin{array}{l}\cdots \\ \dots \end{array}& \begin{array}{l}{w}_{d}^{1}\\ {w}_{d}^{2}\end{array}\\ \begin{array}{cc}\vdots & \vdots \end{array}& \ddots & \vdots \\ \begin{array}{cc}{w}_{1}^{3}& {w}_{2}^{3}\end{array}& \cdots & {w}_{d}^{n}\end{array}\right],$$

where $w$ symbolizes the position of all sharks in the searching space, $d$ indicates the number of a chosen variables for a specific problem.

#### Movement towards prey

White sharks spend the majority of their time seeking and chasing prey because they are creatures with a strong need to survive. They often employ all available tactics to follow and track prey while utilizing their exceptional hearing, sight, and smell skills. A white shark moves to its prey in an undulating motion that may be expressed by Eq. (40) when it locates its prey based on the hesitation of the waves it hears during the movement of the prey.

40 $${v}_{k}^{j}=\mu \left[{v}_{k-1}^{j}+{p}_{1}\left({w}_{gbest,k-1}-{w}_{k}^{j}\right)\times {c}_{1}+{p}_{2}\left({w}_{best}^{{v}_{k-1}^{j}}-{w}_{k-1}^{j}\right)\times {c}_{2}\right],$$

where $j=\text{1,2},\dots ,n$, represents the white shark’s index for $n$ search agents; the new velocity of the *j*th white shark in ${(k-1)}{\text{th}}$ step is denoted by ${v}_{k-1}^{j}$; ${w}_{gbest,k-1}$ is the global best solution obtained so far in the iteration ${(k-1)}{\text{th}}$; ${w}_{best}^{{v}_{k-1}^{j}}$ symbolizes the *j*th best known position for the swarm and ${v}_{k-1}^{j}$ shows the optimal position of white sharks’ index vector, expressed using Eq. (41); ${c}_{1}$ and ${c}_{2}$ are random number between [0,1]; White shark forces governing the influence of ${w}_{gbest,k-1}$ and ${w}_{best}^{{v}_{k-1}^{j}}$ on ${w}_{k-1}^{j}$ are represented by ${p}_{1}$ and ${p}_{2}$, respectively, which are computed by Eqs. (42) and (43); eventually, $\mu$ is the constriction factor to represent the convergence characteristics of the white sharks, formulated as per Eq. (44).

41 $$v=\left[n\times rand(1,n)\right]+1,$$

42 $${p}_{1}={p}_{ub}+\left({p}_{ub}-{p}_{lb}\right)\times {e}^{-{\left(\frac{4a}{A}\right)}^{2}},$$

43 $${p}_{2}={p}_{lb}+\left({p}_{ub}-{p}_{lb}\right)\times {e}^{-{\left(\frac{4a}{A}\right)}^{2}}.$$

Here, $a$ and $A$ are the current and maximum iterations, respectively; ${p}_{lb}$ and ${p}_{ub}$ are respectively the minimum and maximum velocity to accomplish good movement for the white sharks, and their values are 0.5 and 1.5, respectively.

44 $$\mu =\frac{2}{\left|2-\tau -\sqrt{{\tau }^{2}-4\tau }\right|},$$

where, $\tau$ represents the acceleration factor whose value is 4.125.

#### Movement towards optimal prey

Excellent white sharks spend the majority of their time looking for prospective prey, whether the location of the prey is ideal or not. Accordingly, the locations of white sharks are continually varying. When they either hear the waves produced by the motion of prey or detect its scent, they usually proceed toward the prey. In this situation, Eq. (45) illustrates the motion of white sharks as they proceed toward the prey.

45 $${w}_{k}^{j}=\left\{\begin{array}{*{20}l}{w}_{k-1}^{j}\to \oplus {w}_{o}+u.x+l.y & \quad rand<mv\\ {w}_{k-1}^{j}+\frac{{v}_{k-1}^{i}}{f} & \quad rand\ge mv\end{array},\right.$$

where ${w}_{k}^{j}$ represents the new position of the white sharks in the in ${(k-1)}^{th}$ iteration; $x$ and $y$ symbolize one dimensional binary vectors represented by Eqs. (46) and (47); ${w}_{o}$ is a logical vector given by Eq. (48); $\to$ is a negation operator; the lower and upper limits of the search area are represented by $l$ and $u$, respectively; $f$ is the frequency of the wavy movement of the white sharks, which is expressed as per Eq. (49); rand is a random number within the interval [0,1]; finally, the white shark’s movement force, denoted by mv, grows with iteration, as the shark gets closer to its prey, as expressed in Eq. (50).

46 $$x=sgn\left({w}_{k-1}^{j}-u\right)>0,$$

47 $$y=sgn\left({w}_{k-1}^{j}-l\right)<0,$$

48 $${w}_{o}=\oplus \left(x,y\right),$$

where $\oplus$ is a bit-wise XOR operation.

49 $$f={f}_{min}+\frac{{f}_{max}-{f}_{min}}{{f}_{max}+{f}_{min}} .$$

Here, the maximum and minimum frequency of the undulating movement of the white sharks are represented by ${f}_{max}$ and ${f}_{min}$, respectively. Whose values are 0.75 and 0.007, respectively.

50 $$mv=\frac{1}{{d}_{o}+{e}^{(A/2-a)/{d}_{1}}},$$

where ${d}_{o}$ and ${d}_{1}$ are respectively two constant positive numbers that utilized to manage the behaviour of exploration and exploitation phases. $mv$ demonstrates how the white shark’s acute sense of hearing and smell improves with repetition.

#### Movement towards the best white shark

Great white sharks have the ability to sustain their proximity to their best ones that is closer to prey. Equation (51) provides a formalization of this phenomenon.

51 $${\stackrel{`}{w}}_{k}^{j}={w}_{gbest, k-1}+{r}_{1}{\overrightarrow{D}}_{w} sgn\left({r}_{2}-0.5\right) {r}_{3}<s,$$

where ${\stackrel{`}{w}}_{k}^{j}$ is the updated position of the *j*th white sharks with respect to the location of the prey; $sgn\left({r}_{2}-0.5\right)$ provides either 1 or − 1 to reverse the direction of the search; ${r}_{1}$, ${r}_{2}$, and ${r}_{3}$ are normally distributed numbers between [0,1]; the distance between the prey and white shark is illustrated by ${\overrightarrow{D}}_{w}$, as represented by Eq. (52); $s$ demonstrates the efficacy of olfaction and vision in white sharks as they track other white sharks that are in proximity to ideal food, which is formulated as given in Eq. (53).

52 $${\overrightarrow{D}}_{w}=\left|rand\times \left({w}_{gbest, k-1}-{w}_{k-1}^{j}\right)\right|,$$

where $rand$ is a random number within the range [0,1]; ${w}_{k-1}^{j}$ represents the current position of the white shark in respect to the best position, ${w}_{gbest, k-1}$.

53 $$s=\left|1-{e}^{-\left(ga/A\right)}\right| ,$$

where $g$ is a positive constant number, whose value is 0.0005 to manage the behaviour of exploration and exploitation stages.

#### Fish school behavior

The first two ideal candidates were kept, and the location of other white sharks was modified in accordance with these optimal positions to mathematically imitate the behavior of the school of white sharks. The following formula is presented to characterize white shark schooling behavior:

54 $${w}_{k}^{j}=\frac{{w}_{k-1}^{j}+{\stackrel{`}{w}}_{k}^{j}}{2rand}.$$

**Figure 5:**
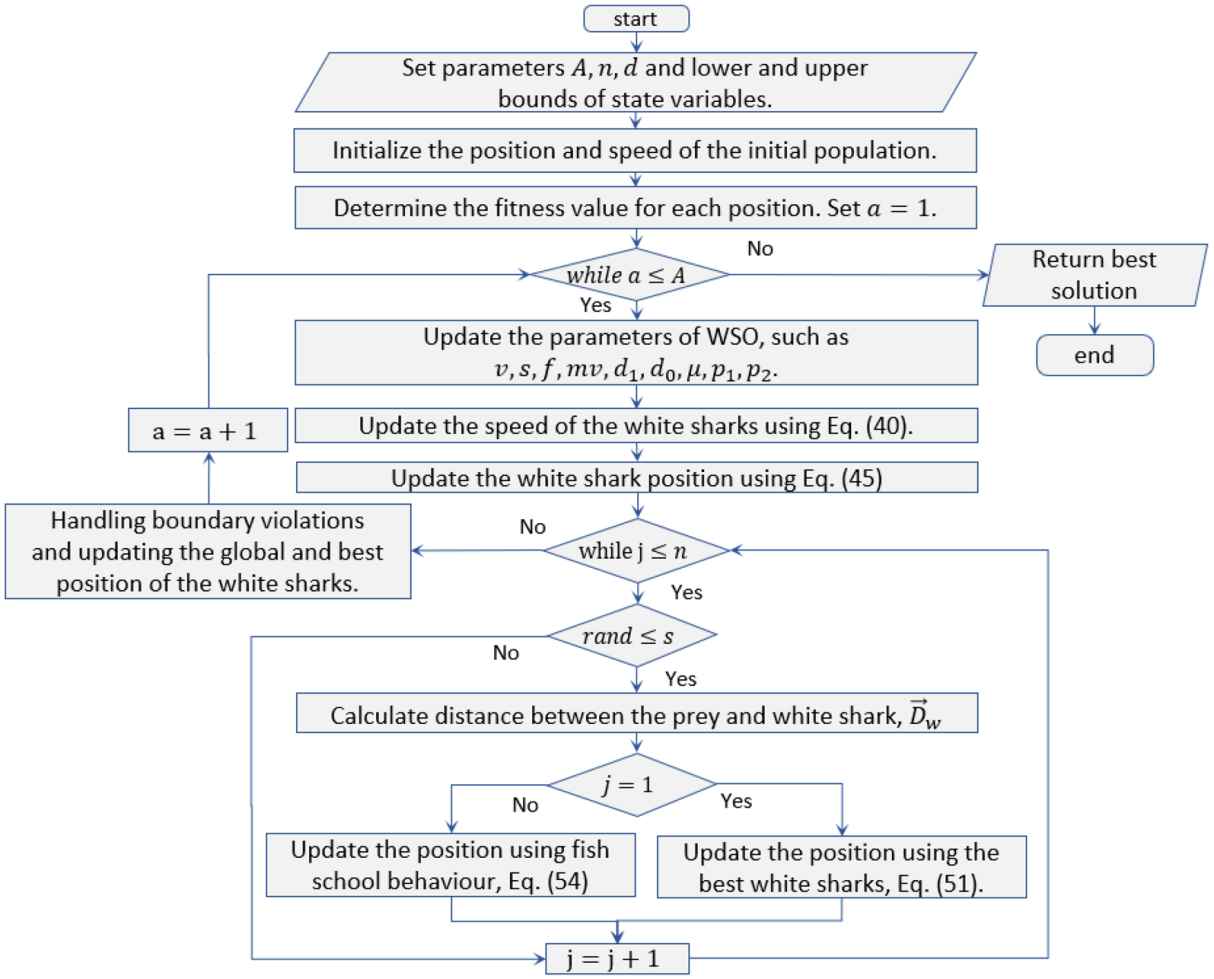
The complete flowchart of the original WSO algorithm.

Equation (54) shows that white sharks may adjust their location such that it matches that of the best white shark, which has now moved into an ideal location, extremely near to its meal. Great white sharks (i.e., search agents) would end up in a location in the search space that is almost ideal for their prey. Collective actions, such as schooling fish behavior or white sharks migrating to find the best white shark, are indicative of WSO and expand the potential for more fruitful exploration and exploitation. The complete flowchart for the original WSO algorithm is represented in Fig. 5.

### Modified WSO algorithm

**Figure 6:**
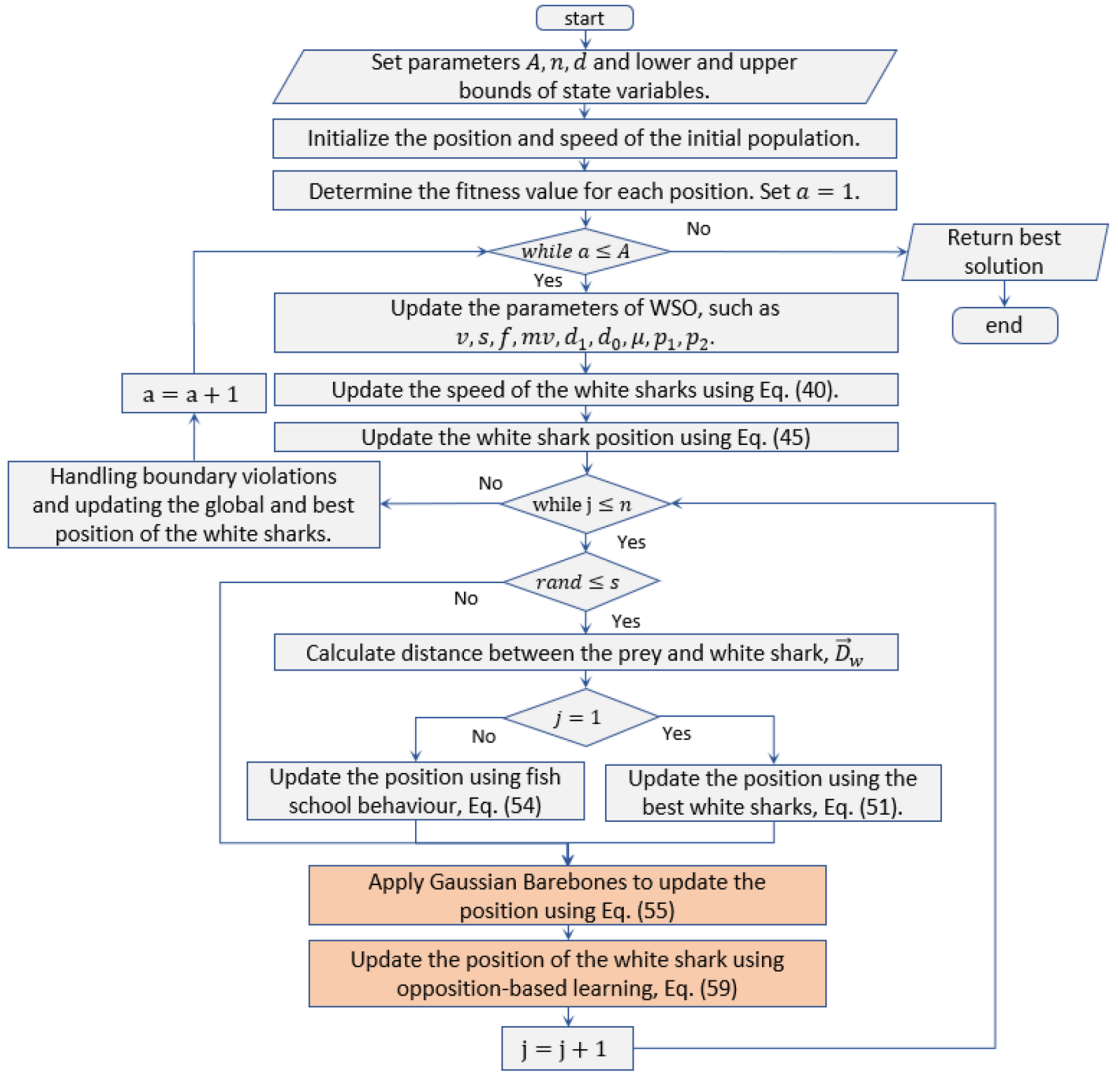
The complete flowchart of the proposed MWSO algorithm.

In the original form of the WSO, the great white sharks move toward their prey spot using a single approach, which may cause the algorithm to miss additional favorable positions in the surrounding area. In other words, the white shark optimization (WSO) algorithm tends to lose diversity as its evaluation progresses, which can cause challenges with convergence speed and accuracy. Additionally, the WSO has been applied for solving some of the complex optimization challenges as reported in “Introduction” section; however, it has some limitations, such as unbalanced exploration and exploitation, a propensity to become stuck in suboptimal local areas, and a sluggish convergence speed. Therefore, in this study, a new version of the WSO is introduced to overcome these issues, which incorporates the Gaussian barebones (GB) and opposition-based learning (QOBL) strategies. The developed MWSO algorithm is exhibited as follows: Initially, the population is randomly generated, and the optimal individual in the current individual is identified based on the objective function. Then, update the position of the white sharks using Eqs. (51) and (54). The GB is adopted to find superior positions for the updated white sharks. Eventually, the QOBL mechanism is employed to update the individuals again. The increasing diversity of the population improves the exploitation ability and enhances the convergence speed and accuracy of the MWSO algorithm. The strategies of the GB and QOBL are illustrated in the following subsections. Table 1 exhibits the complete pseudo code of the proposed MWSO. Eventually, Fig. 6 shows the complete flowchart of the proposed MWSO algorithm.

**Table 1:**
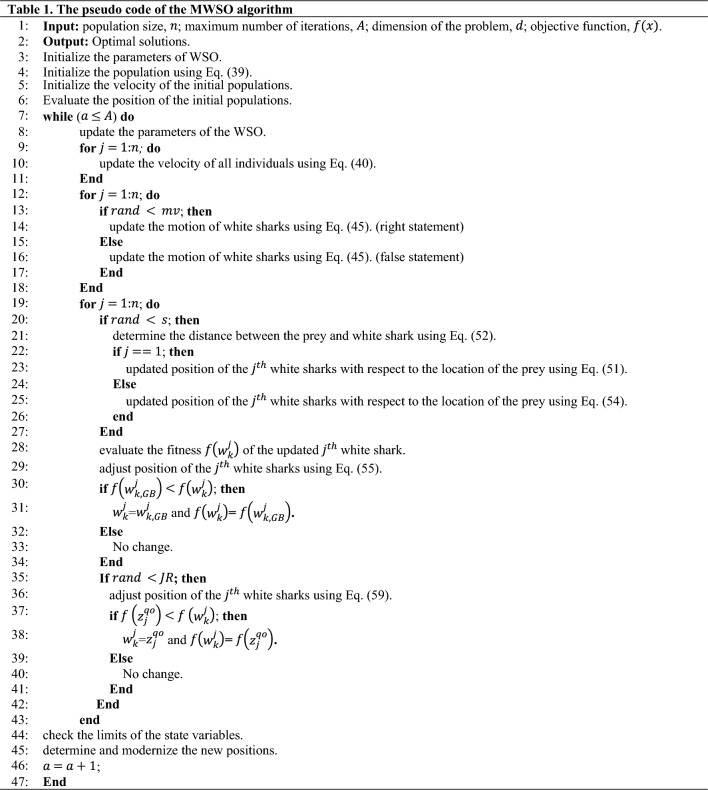
The pseudo code of the MWSO algorithm.

#### Gaussian barebones strategy

As previously stated, during the later phase of evaluating the WSO algorithm, the variety of the white shark optimization (WSO) algorithm diminishes, leading to potential issues with convergence speed and accuracy. The GB strategy facilitates the selection of the most suitable direction for white sharks and enables them to steadily progress towards the optimal solution, hence preventing premature convergence to local optima. Consequently, once the positions of all search agents have been updated, the inclusion of GB’s randomization features into WSO is employed to augment the variety of the population. This maintains a balance between the algorithm’s local exploitation and its capability for global search, resulting in enhanced convergence speed.

The GB strategy is derived from the bare-bones PSO (BBPSO) algorithm^57^. In this strategy, the parameter R is utilized to guide each individual. If the chance of random generation is less than R, the individual’s location is updated using the Gaussian distribution in the next assessment; alternatively, the concept of differential evolution is included. Eventually, the GB strategy may be expressed as follows:

55 $${w}_{k,GB}^{j}=\left\{\begin{array}{*{20}l}G\left(\frac{\left({w}_{gbest, k-1}+{w}_{k}^{j}\right)}{2},\left|{w}_{gbest, k-1}-{w}_{k}^{j}\right|\right) &\quad if \,rand<R\\ {w}_{k}^{j1}+{r}_{4}\left({w}_{k}^{j2}-{w}_{k}^{j3}\right)& \quad otherwise\end{array}\right.,$$

where ${w}_{k,GB}^{j}$ represents the new position for the *j*th white shark using the GB mechanism; ${w}_{gbest, k-1}$ symbolizes the global best solution obtained so far in the iteration ${(k-1)}{th}$; ${r}_{4}$ is random number within the interval [0,1]; $G$ denotes the Gaussian distribution with mean $\left(\left({w}_{gbest, k-1}+{w}_{k}^{j}\right)/2\right)$ and standard deviation $\left({w}_{gbest, k-1}-{w}_{k}^{j}\right)$; $j1$, $j2$, and $j3$ are three randomly chosen individuals that are diverse from the current individual, $j$; ${w}_{k}^{j}$ is the updated position of *j*th white shark using fish school behaviour in the current iteration *k*th.

#### Quasi-oppositional movement strategy

The opposition-based learning (OBL) method, initially proposed by Tizhoosh^58^, can speed up convergence and boost solution quality by simultaneously considering both the current solution and the exact opposite one. According to probability theory, each answer has a 50% chance of being better than the other. The best of the two inverse solutions is picked as the candidate solution to improve the evolutionary algorithms’ search efficiency. The OBL approach has been successfully used across a wide range of challenges. Definitions of opposing numbers and opposite points are provided in this work^59^ to help clarify the notion of opposition-based learning.

##### Opposite number

If $z$ is a random number in the range $[{a}_{1}, {a}_{2}]$, then its opposite one may be written as:

56 $${z}^{o}={a}_{1}+{a}_{2}-z.$$

##### Opposite point

If $p({z}_{1}, {z}_{2},\dots ,{z}_{j},\dots ,{z}_{d})$ is a point in a searching space with a $d$-dimensional in which the ${z}_{j}\in [{a}_{1,j}, {a}_{2,j}]$, then its opposite one $op({z}_{1}^{o}, {z}_{2}^{o},\dots ,{z}_{j}^{o},\dots ,{z}_{d}^{o})$ is expressed by the following relation:

57 $${z}_{j}^{o}={a}_{1,j}+ {a}_{2,j}-{z}_{j}.$$

Nevertheless, it’s worth noting that OBL has certain development processes and that several researches have demonstrated that quasi-opposition-based learning (QOBL) is more successful than OBL^60^. So, the definitions of the quasi-opposite number and point are as below:

##### Quasi-opposite number

A random number $z$ in the search region $[{a}_{1}, {a}_{2}]$ has a quasi-opposite number ${z}^{qo}$, which may be written as.

58 $${z}^{qo}=rand\left[\left(\frac{{a}_{1}+{a}_{2}}{2}\right),\left({a}_{1}+{a}_{2}-z\right)\right].$$

##### Quasi-opposite point

Similarly, the quasi-opposite point, $qop({z}_{1}^{qo}, {z}_{2}^{qo},\dots ,{z}_{j}^{qo},\dots ,{z}_{d}^{qo})$ in a searching region with a $d$-dimensional problem is calculated as per Eq. (59).

59 $${z}_{j}^{qo}=rand\left[\left(\frac{{a}_{1,j}+{a}_{2,j}}{2}\right),\left({a}_{1,j}+{a}_{2,j}-{z}_{j}\right)\right].$$

The QOBL methodology may be utilized not only during the initialization phase but also throughout the evolutionary phase of a WSO algorithm for updating the individuals. In the current study, the solution obtained by the Gaussian barebones process utilizing Eq. (55) has the potential to be substituted with a quasi-opposite solution.

## Simulation results and discussion

In this section, the performance of the MWSO algorithm is evaluated and quantified using the 23rd standard benchmark functions. These benchmark functions comprise seven unimodal functions, six high-dimensional multi-modal functions, and eight fixed high-dimensional multi-modal functions. Furthermore, the application of the MWSO algorithm is applied to solving the optimal power flow of the modified IEEE 30-bus and 57-bus power systems, considering several real-world scenarios. In this study, known metaheuristic algorithms like SSA, PSO, NOA, KOA, and WOA, as well as the original WSO are utilized to evaluate MWSO’s performance. In this situation, the default parameters of these rivals are utilized as recommended by the designer of the algorithms. For a fair comparison of outcomes, each algorithm is performed thirty times, with a population size of 30 and a maximum number of iterations of 1000. Several statistical metrics, such as best, mean, standard deviation, rank, and Wilcoxon rank-sum p-values are employed in this study. Specifically, “Windows 10 (64bit)” is used for the OS, “CPU Core i7 with 16 GB of RAM” is the hardware setup, and “MatLab 2020b” is the analytical tool of choice.

### Experimental results of CEC 2017 testing functions

#### Mathematical validation

The improved WSO method demonstrates superior performance in finding optimal solutions for unimodal functions, namely F1, F3, F4, and F7, beating the competing optimization algorithms, as seen in Table 2. In other words, the MWSO algorithm surpasses the original algorithm in these test functions. Conversely, the KOA method exhibits the most unfavourable results. Concerning the type of high-dimensional multimodal test functions, namely F8 through F13, the MWSO algorithm tends to get trapped in local optimum solutions for F8, F12, and F13. The modified MWSO algorithm demonstrates superior performance compared to the standard WSO and other rivals in achieving global solutions for various fixed multimodal testing functions i.e., F14–F23. Furthermore, it can be noticed that the MWSO algorithm offers the best overall rank, exceeding all the efficient and recent competing algorithms. Therefore, the findings concluded that the MWSO algorithm yields improved outcomes in tackling such optimization issues.

**Table 2:** Results of the MWSO and comparison against other competitors on the CEC2017 benchmark.

F(x)	Index	MWSO	WSO	SSA	NOA	KOA	PSO	WOA
F1	Min	**1.9932e**−**194**	3.6522e+00	7.6851e−09	3.9820e+04	3.5508e+04	2.6735e−06	2.4631e−169
	Avg	**7.4390e**−**186**	1.6060e+01	1.2920e−08	5.4068e+04	4.2092e+04	3.9912e−05	2.0743e−148
	Std	**0.0000e+00**	1.0322e+01	3.3486e−09	4.4121e+03	3.1944e+03	6.8864e−05	1.1316e−147
	Mean rank	**1.0000e+00**	5.0000e+00	3.0000e+00	7.0000e+00	6.0000e+00	4.0000e+00	2.0000e+00
F2	Min	9.4274e−98	2.9819e−01	5.1425e−03	2.1049e+05	7.8389e+01	1.3092e−03	**1.1534e**−**116**
	Avg	4.8694e−94	6.2085e−01	1.0820e+00	1.0628e+09	1.2886e+06	6.7182e−03	**1.4894e**−**104**
	Std	1.1095e−93	2.2835e−01	1.1441e+00	3.0026e+09	2.0077e+06	5.9536e−03	**5.0498e**−**104**
	Mean rank	2.0000e+00	4.5333e+00	4.4000e+00	6.9333e+00	6.0667e+00	3.0667e+00	**1.0000e+00**
F3	Min	**6.5221e**−**145**	1.8779e+02	5.5282e+01	4.1463e+04	4.0477e+04	5.8665e+00	2.4173e+03
	Avg	**2.8306e**−**130**	6.9505e+02	2.6853e+02	7.4458e+04	5.0203e+04	2.1389e+01	2.1307e+04
	Std	**1.3850e**−**129**	3.3974e+02	1.9808e+02	1.1229e+04	5.2022e+03	9.0540e+00	9.3229e+03
	Mean rank	**1.0000e+00**	3.9000e+00	3.1000e+00	6.9333e+00	6.0667e+00	2.0000e+00	5.0000e+00
F4	Min	**1.9845e**−**92**	7.3432e+00	2.8883e+00	7.1797e+01	6.4039e+01	4.1628e−01	4.4710e−02
	Avg	**1.9938e**−**88**	1.2150e+01	8.3506e+00	8.0033e+01	7.1198e+01	6.8292e−01	3.8983e+01
	Std	**5.2424e**−**88**	2.6366e+00	3.5469e+00	2.3644e+00	2.7995e+00	1.7217e−01	2.7709e+01
	Mean rank	**1.0000e+00**	4.0333e+00	3.4000e+00	6.9333e+00	5.8333e+00	2.1000e+00	4.7000e+00
F5	Min	2.7223e+01	1.6931e+02	2.4671e+01	7.5841e+07	6.7063e+07	**1.8634e+01**	2.6279e+01
	Avg	2.8228e+01	1.2400e+03	1.0650e+02	1.5660e+08	1.0843e+08	9.4003e+01	**2.7082e+01**
	Std	**2.8797e**−**01**	1.6683e+03	1.3804e+02	2.3965e+07	1.6971e+07	1.0055e+02	4.0881e−01
	Mean rank	2.5667e+00	4.9000e+00	3.0000e+00	6.9667e+00	6.0333e+00	3.1000e+00	**1.4333e+00**
F6	Min	4.2641e−03	4.2166e+00	**6.9384e**−**09**	4.1142e+04	3.4416e+04	2.2344e−06	7.6167e−03
	Avg	3.1379e−02	1.7249e+01	**1.2490e**−**08**	5.1496e+04	4.2640e+04	1.9953e−05	9.5573e−02
	Std	2.7474e−02	1.1310e+01	**2.6446e**−**09**	4.2733e+03	3.2831e+03	2.2893e−05	1.1334e−01
	Mean rank	3.2333e+00	5.0000e+00	**1.0000e+00**	6.9333e+00	6.0667e+00	2.0000e+00	3.7667e+00
F7	Min	**3.3636e**−**05**	2.6410e−02	4.2938e−02	3.8762e+01	2.7258e+01	2.6260e−02	7.0396e−05
	Avg	**3.8968e**−**04**	1.2871e−01	9.0210e−02	7.1476e+01	4.9218e+01	7.8548e−02	1.9816e−03
	Std	**2.8817e**−**04**	6.1893e−02	3.3215e−02	1.2870e+01	6.9079e+00	2.9457e−02	2.4388e−03
	Mean rank	**1.2000e+00**	4.5333e+00	3.8667e+00	6.9000e+00	6.1000e+00	3.6000e+00	1.8000e+00
F8	Min	− 1.1856e+04	− 8.6242e+03	− 8.7363e+03	− 5.4177e+03	− 5.4177e+03	− 7.8120e+03	− **1.2569e+04**
	Avg	− 1.0282e+04	− 6.6137e+03	− 7.4827e+03	− 5.4177e+03	− 5.4177e+03	− 6.3816e+03	− **1.1183e+04**
	Std	8.9674e+02	1.2738e+03	5.8567e+02	**1.8501e**−**12**	**1.8501e**−**12**	7.8520e+02	1.6926e+03
	Mean rank	1.7667e+00	4.4333e+00	3.3000e+00	6.2333e+00	6.2333e+00	4.7333e+00	**1.3000e+00**
F9	Min	**0.0000e+00**	1.1067e+01	2.0894e+01	3.5064e+02	3.0942e+02	3.1958e+01	0.0000e+00
	Avg	**0.0000e+00**	1.9154e+01	5.6845e+01	3.8445e+02	3.4114e+02	5.4736e+01	1.8948e−15
	Std	**0.0000e+00**	5.5047e+00	1.8204e+01	1.3984e+01	1.4048e+01	1.7115e+01	1.0378e−14
	Mean rank	**1.4833e+00**	3.0333e+00	4.5333e+00	7.0000e+00	6.0000e+00	4.4333e+00	1.5167e+00
F10	Min	**8.8818e**−**16**	1.6786e+00	3.3794e−05	1.9546e+01	1.9669e+01	1.2149e−03	8.8818e−16
	Avg	**8.8818e**−**16**	3.4203e+00	2.2748e+00	1.9953e+01	1.9910e+01	2.6531e−02	4.2040e−15
	Std	**0.0000e+00**	7.4273e−01	7.0387e−01	7.6784e−02	8.4780e−02	1.1697e−01	2.2726e−15
	Mean rank	**1.1167e+00**	4.9333e+00	4.0333e+00	6.7333e+00	6.2667e+00	3.0333e+00	1.8833e+00
F11	Min	**0.0000e+00**	1.0385e+00	4.6605e−08	3.9589e+02	3.3058e+02	2.3021e−07	**0.0000e+00**
	Avg	**0.0000e+00**	1.1709e+00	7.6302e−03	4.6440e+02	3.8146e+02	1.2392e−02	1.0628e−02
	Std	**0.0000e+00**	9.8272e−02	9.2033e−03	3.9991e+01	3.0929e+01	1.2621e−02	2.9724e−02
	Mean rank	**1.4333e+00**	5.0000e+00	3.0667e+00	6.9333e+00	6.0667e+00	3.6667e+00	1.8333e+00
F12	Min	7.6123e−04	4.1084e−01	1.5302e+00	2.2733e+08	7.3617e+07	**1.5069e**−**08**	4.6340e−04
	Avg	4.6373e−03	1.5823e+00	5.5578e+00	3.3847e+08	1.8705e+08	**3.4563e**−**03**	5.0799e−03
	Std	**2.9583e**−**03**	9.5940e−01	2.6552e+00	6.2117e+07	3.9501e+07	1.8927e−02	5.8770e−03
	Mean rank	2.5333e+00	4.0000e+00	5.0000e+00	7.0000e+00	6.0000e+00	**1.0667e+00**	2.4000e+00
F13	Min	3.7409e−03	3.2200e+00	**1.1488e**−**09**	2.4495e+08	2.6676e+08	4.6491e−07	2.4791e−02
	Avg	9.3988e−02	1.7006e+01	8.3911e−01	6.6291e+08	4.2446e+08	**2.2059e**−**03**	2.5688e−01
	Std	8.7197e−02	8.7602e+00	3.1879e+00	1.4737e+08	7.1767e+07	**4.4681e**−**03**	1.6126e−01
	Mean rank	3.0667e+00	4.9667e+00	1.6667e+00	6.9000e+00	6.1000e+00	**1.5333e+00**	3.7667e+00
F14	Min	**9.9800e**−**01**	**9.9800e**−**01**	**9.9800e**−**01**	**9.9869e**−**01**	**9.9803e**−**01**	**9.9800e**−**01**	**9.9800e**−**01**
	Avg	**9.9800e**−**01**	**9.9800e**−**01**	**9.9800e**−**01**	4.0327e+00	1.2030e+00	4.5458e+00	1.8527e+00
	Std	6.4730e−07	**0.0000e+00**	2.3142e−16	2.0552e+00	4.4364e−01	2.5079e+00	1.8844e+00
	Mean rank	2.2667e+00	**1.3000e+00**	2.6500e+00	6.2667e+00	4.8333e+00	6.1500e+00	4.5333e+00
F15	Min	**3.0749e**−**04**	**3.0749e**−**04**	3.8664e−04	3.5648e−03	7.9158e−04	3.2718e−04	3.0760e−04
	Avg	1.1765e−03	**3.5037e**−**04**	1.4680e−03	1.9990e−02	3.0550e−03	8.1635e−04	7.3231e−04
	Std	3.6341e−03	**2.3489e**−**04**	3.5778e−03	1.0115e−02	1.6101e−03	2.6038e−04	4.5385e−04
	Mean rank	2.6333e+00	**1.3000e+00**	3.9667e+00	6.9333e+00	5.8667e+00	3.9667e+00	3.3333e+00
F16	Min	− **1.0316e+00**	− **1.0316e+00**	− **1.0316e+00**	− 1.0289e+00	− **1.0316e+00**	− **1.0316e+00**	− **1.0316e+00**
	Avg	− **1.0316e+00**	− **1.0316e+00**	− **1.0316e+00**	− 9.3965e−01	− 1.0254e+00	− **1.0316e+00**	− **1.0316e+00**
	Std	6.7752e−16	5.5296e−08	8.2402e−15	7.9640e−02	5.1597e−03	**6.6486e**−**16**	1.2296e−10
	Mean rank	**1.8833e+00**	2.3000e+00	3.9333e+00	6.9667e+00	6.0333e+00	1.9833e+00	4.9000e+00
F17	Min	**3.9789e**−**01**	**3.9789e**−**01**	**3.9789e**−**01**	4.0288e−01	3.9801e−01	**3.9789e**−**01**	**3.9789e**−**01**
	Avg	**3.9789e**−**01**	**3.9789e**−**01**	**3.9789e**−**01**	4.7261e−01	4.0088e−01	**3.9789e**−**01**	**3.9789e**−**01**
	Std	**0.0000e+00**	1.5223e−05	1.4947e−14	8.0451e−02	2.2983e−03	**0.0000e+00**	2.2220e−06
	Mean rank	**1.9500e+00**	2.5000e+00	3.7333e+00	7.0000e+00	6.0000e+00	**1.9500e+00**	4.8667e+00
F18	Min	**3.0000e+00**	**3.0000e+00**	**3.0000e+00**	3.0175e+00	3.0025e+00	**3.0000e+00**	**3.0000e+00**
	Avg	**3.0000e+00**	**3.0000e+00**	**3.0000e+00**	6.2793e+00	3.1109e+00	**3.0000e+00**	**3.0000e+00**
	Std	**1.2148e**−**15**	1.3065e−15	8.4529e−14	3.7125e+00	1.2168e−01	1.7494e−15	6.5496e−06
	Mean rank	**1.7500e+00**	1.9833e+00	4.0000e+00	6.8667e+00	6.1333e+00	2.2667e+00	5.0000e+00
F19	Min	− **3.8628e+00**	− **3.8628e+00**	− **3.8628e+00**	− 3.8578e+00	− 3.8610e+00	− **3.8628e+00**	− **3.8628e+00**
	Avg	− **3.8628e+00**	− **3.8628e+00**	− **3.8628e+00**	− 3.7959e+00	− 3.8552e+00	− **3.8628e+00**	− 3.8615e+00
	Std	**2.7101e**−**15**	**2.7101e**−**15**	3.6715e−14	5.9794e−02	6.2723e−03	**2.7101e**−**15**	1.5224e−03
	Mean rank	**2.0000e+00**	**2.0000e+00**	4.0000e+00	6.9333e+00	5.9333e+00	**2.0000e+00**	5.1333e+00
F20	Min	− **3.3220e+00**	− **3.3220e+00**	− **3.3220e+00**	− 3.1574e+00	− 3.1910e+00	− **3.3220e+00**	− **3.3220e+00**
	Avg	− 3.2824e+00	− **3.3141e+00**	− 3.2115e+00	− 2.6353e+00	− 3.0313e+00	− 3.2744e+00	− 3.2668e+00
	Std	5.7005e−02	**3.0164e**−**02**	3.7688e−02	2.4846e−01	7.1081e−02	5.9241e−02	8.1351e−02
	Mean rank	2.4667e+00	**1.6833e+00**	4.5000e+00	6.9000e+00	6.0667e+00	2.5500e+00	3.8333e+00
F21	Min	− **1.0153e+01**	− **1.0153e+01**	− **1.0153e+01**	− 6.6251e+00	− 7.0479e+00	− **1.0153e+01**	− **1.0153e+01**
	Avg	− **1.0153e+01**	− 9.6552e+00	− 8.0541e+00	− 1.6617e+00	− 3.7081e+00	− 7.3121e+00	− 8.6261e+00
	Std	**7.2269e**−**15**	1.8953e+00	2.8837e+00	1.2534e+00	1.5068e+00	3.3991e+00	2.6208e+00
	Mean rank	**1.5167e+00**	1.8667e+00	3.9667e+00	6.7333e+00	5.8000e+00	3.7167e+00	4.4000e+00
F22	Min	− **1.0403e+01**	− **1.0403e+01**	− **1.0403e+01**	− 5.6447e+00	− 7.1042e+00	− **1.0403e+01**	− **1.0403e+01**
	Avg	− **1.0403e+01**	− 9.8287e+00	− 8.6141e+00	− 1.7158e+00	− 3.8817e+00	− 9.1114e+00	− 8.5318e+00
	Std	**1.5472e**−**15**	1.7650e+00	2.8346e+00	8.6329e−01	1.2337e+00	2.6819e+00	3.2241e+00
	Mean rank	**1.4333e+00**	2.1500e+00	4.1000e+00	6.8667e+00	5.7667e+00	3.0500e+00	4.6333e+00
F23	Min	− **1.0536e+01**	− **1.0536e+01**	− **1.0536e+01**	− 3.3045e+00	− 5.2821e+00	− **1.0536e+01**	− **1.0536e+01**
	Avg	− **1.0536e+01**	− **1.0536e+01**	− 9.0693e+00	− 1.8972e+00	− 3.3515e+00	− 9.7433e+00	− 8.3397e+00
	Std	**1.8067e**−**15**	1.8949e−15	2.7594e+00	6.0525e−01	7.7805e−01	2.0894e+00	2.9624e+00
	Mean rank	**1.8167e+00**	1.8667e+00	4.2000e+00	6.9000e+00	5.9000e+00	2.5500e+00	4.7667e+00
Overall mean rank		**1.8746e+00**	3.3572e+00	3.5833e+00	6.8594e+00	5.9638e+00	2.9790e+00	3.3826e+00
Overall rank		**1**	3	5	7	6	2	4

*Bold face highlights the best obtained solutions.

### Convergence curve

**Figure 7:**
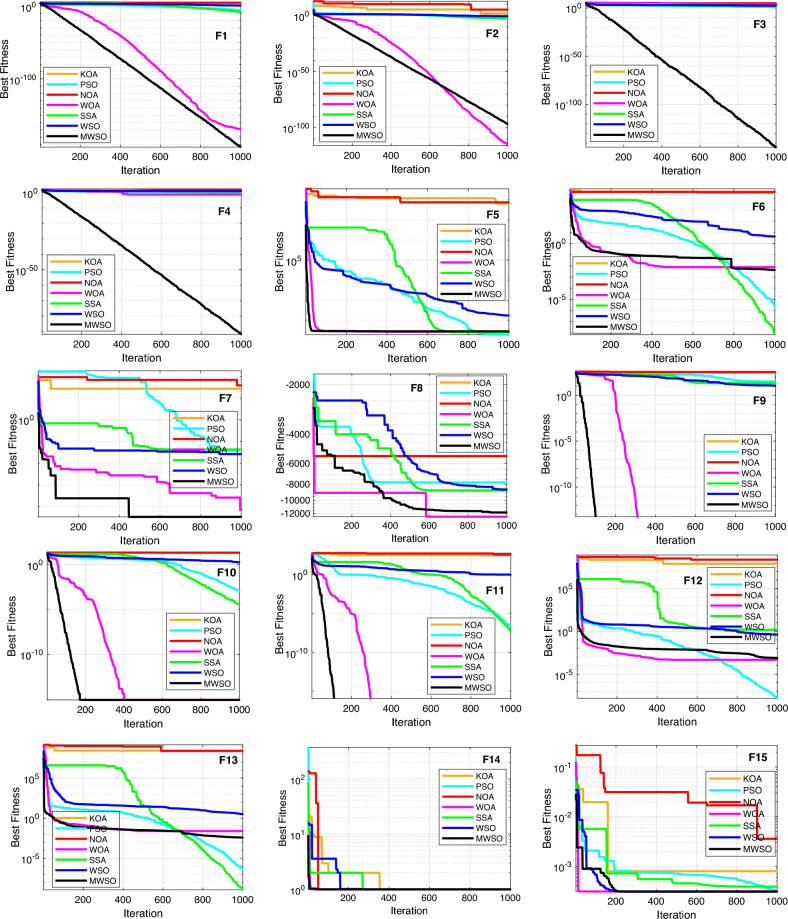

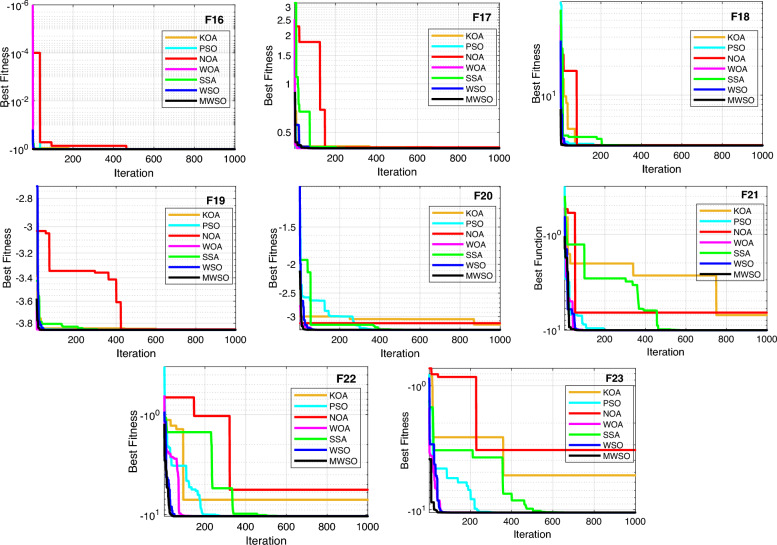
Convergence characteristics of the developed HRSOAPOA and other competitors for CEC 2017 benchmark functions.

With a dimension of 30 for the unimodal and multimodal functions, Fig. 7 visually analyzes the convergence rate of the developed MWSO method across the CEC 2017 benchmark testing functions. As can be observed in this figure, the proposed algorithm converges more quickly than other methods, particularly for the functions F1, F3, F7, F4, F9, F10, and F11. The functions F2, F6, and F13 are examples of situations in which MWSO becomes trapped in a nearly optimal state. Additionally, the developed algorithm exceeds the original one for evaluating the best optimal solutions for all testing functions. In any case, the modified optimizer often yields better results with fewer iterations. Due to its quick convergence and improved accuracy, the MWSO approach is an efficient optimization tool for handling increasingly complex optimization scenarios.

### Boxplot behaviour

**Figure 8:**
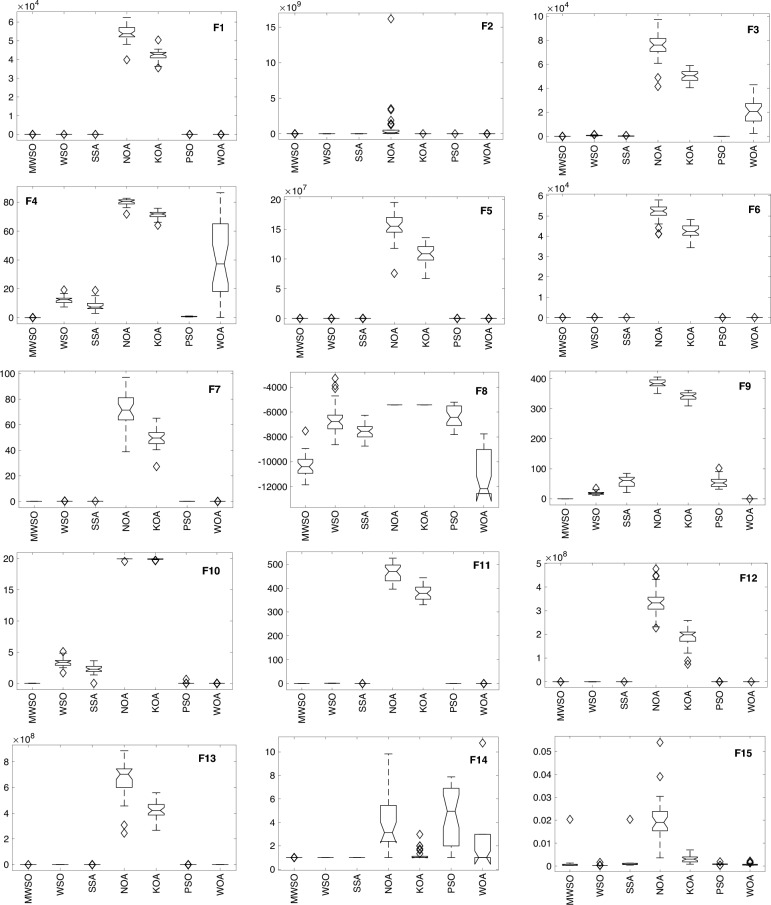

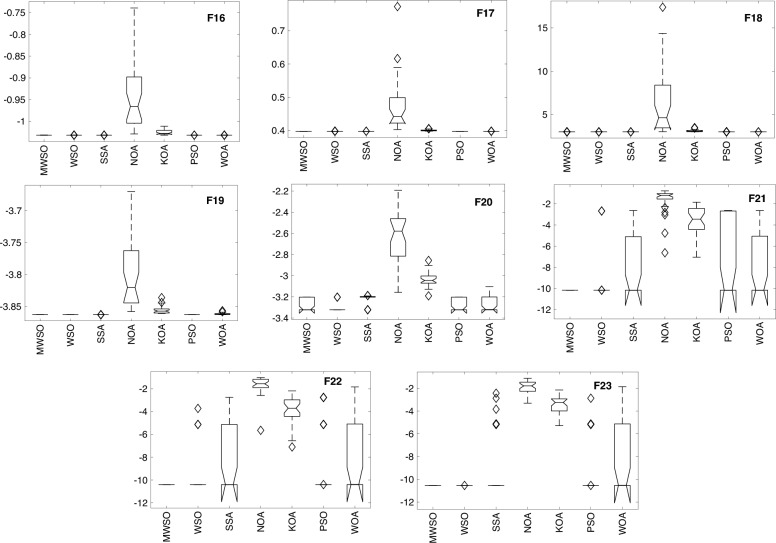
Box plot of the modified MWSO and other rivals 23rd testing functions**.**

Figure 8 depicts the boxplot curves derived from the MWSO optimizer and its competing counterparts. The figure visually represents the distribution of data across all functions from the CEC 2017 dataset. The whiskers on the boxplots represent the minimum and maximum values achieved by the algorithms. A tight box plot is indicative of a significant level of data consensus. Specifically, the MWSO method exhibits a lack of outliers throughout a set of more than ten functions, namely F7, F9, F10, F11, F16, F17, F19, F21, F22, and F23. Upon evaluating the boxplots of the majority of the testing functions, it becomes evident that the MWSO optimizer has a superior distribution characterized by lower values. The MWSO approach continuously exhibits better performance compared to other existing optimization methods, hence confirming its enhanced usefulness.

### p-value-based statistical analysis

The statistical significance of the findings acquired by the MWSO algorithm and other competing algorithms is assessed via the use of the Wilcoxon rank-sum test. This test is employed to demonstrate that the observed performance was not only attributable to random chance. The analysis is carried out using a substantial threshold of 5% for all testing functions. Table 3 presents a summary of the outcomes obtained from the MWSO algorithm compared to the competing algorithms, as evaluated by the Wilcoxon test. It can be noticed from this table that the developed algorithm differs significantly from the other optimizers, in which the p-value is less than the significance level of 0.05, indicating that MWSO outperforms the original WSO and the others in terms of attaining optimum solutions and a higher convergence rate. Furthermore, the results of the 23 functions are shown in Table 4, using ANOVA analysis, the Friedman test, and the Kruskal test. According to the table, the MWSO demonstrates significantly greater efficacy in comparison to the six competing alternatives, as shown by p-values below 0.05.

**Table 3:** Wilcoxon Rank test of MWSO vs compared methods for CEC2017.

F(x)	WSO	SSA	NOA	KOA	WOA	PSO
F1	3.0199e−11	3.0199e−11	3.0199e−11	3.0199e−11	3.0199e−11	3.0199e−11
F2	3.0199e−11	3.0199e−11	3.0199e−11	3.0199e−11	3.0199e−11	3.0199e−11
F3	3.0199e−11	3.0199e−11	3.0199e−11	3.0199e−11	3.0199e−11	3.0199e−11
F4	3.0199e−11	3.0199e−11	3.0199e−11	3.0199e−11	3.0199e−11	3.0199e−11
F5	3.0199e−11	3.9167e−02	3.0199e−11	3.0199e−11	1.3289e−10	3.9881e−04
F6	3.0199e−11	3.0199e−11	3.0199e−11	3.0199e−11	1.6955e−02	3.0199e−11
F7	3.0199e−11	3.0199e−11	3.0199e−11	3.0199e−11	3.9881e−04	3.0199e−11
F8	5.4941e−11	1.4643e−10	1.2118e−12	1.2118e−12	4.2259e−03	3.6897e−11
F9	1.2118e−12	1.2118e−12	1.2118e−12	1.2118e−12	3.3371e−01	1.2118e−12
F10	1.2118e−12	1.2118e−12	2.7085e−14	8.3126e−13	3.0610e−09	1.2118e−12
F11	1.2118e−12	1.2118e−12	1.2118e−12	1.2118e−12	4.1926e−02	1.2118e−12
F12	3.0199e−11	3.0199e−11	3.0199e−11	3.0199e−11	3.7108e−01	5.5727e−10
F13	3.0199e−11	2.0283e−07	3.0199e−11	3.0199e−11	7.2208e−06	5.4941e−11
F14	1.2499e−05	6.1867e−02	2.0655e−11	2.0655e−11	2.1672e−08	1.3755e−09
F15	3.4448e−04	1.6775e−04	1.7656e−10	1.8462e−09	2.6055e−02	5.2511e−05
F16	2.1577e−02	1.2009e−12	1.2118e−12	1.2118e−12	1.2118e−12	1.6074e−01
F17	2.1577e−02	1.6212e−11	1.2118e−12	1.2118e−12	1.2118e−12	NaN
F18	1.0708e−01	7.8455e−12	7.8455e−12	7.8455e−12	7.8455e−12	2.6002e−03
F19	NaN	1.2039e−12	1.2118e−12	1.2118e−12	1.2118e−12	NaN
F20	1.7183e−03	6.5405e−10	2.1073e−11	2.1073e−11	2.0080e−04	5.2916e−01
F21	4.1896e−02	1.2118e−12	1.2118e−12	1.2118e−12	1.2118e−12	5.2781e−10
F22	6.2298e−03	8.8675e−12	8.8675e−12	8.8675e−12	8.8675e−12	1.9068e−08
F23	3.3371e−01	1.2118e−12	1.2118e−12	1.2118e−12	1.2118e−12	6.5598e−04

**Table 4:** Outcomes from the ANOVA, Friedman, and Kruskal tests.

	F1	F2	F3	F4	F5	F6
p-value based Friedman	3.3931e−36	4.3111e−35	8.8349e−36	1.0967e−32	5.1648e−31	1.5447e−35
p-value based ANOVA	1.5463e−207	1.4395e−03	7.4067e−138	8.5406e−98	1.8352e−150	3.2403e−206
p-value based Kruskal	2.2729e−41	2.0531e−40	5.6156e−41	2.0379e−37	8.6052e−36	1.4496e−40

	F7	F8	F9	F10	F11	F12
p-value based Friedman	2.0434e−33	1.3308e−30	2.3827e−35	1.3791e−35	3.2198e−34	2.9161e−35
p-value based ANOVA	6.2930e−143	2.9250e−78	4.5341e−222	1.1788e−270	1.8781e−202	2.4719e−133
p-value based Kruskal	3.0419e−38	3.8606e−35	9.8865e−41	9.9904e−41	3.2541e−39	3.0012e−40

	F13	F14	F15	F16	F17	F18
p-value based Friedman	9.4219e−34	1.1191e−29	1.3047e−27	1.6469e−34	6.8706e−34	1.1501e−34
p-value based ANOVA	2.5830e−125	7.8165e−29	2.6467e−51	2.0627e−31	1.5836e−22	9.5108e−21
p-value based Kruskal	9.7793e−39	7.1766e−34	2.5737e−31	1.3765e−39	6.4209e−39	1.1556e−39

	F19	F20	F21	F22	F23	
p-value based Friedman	1.2007e−35	4.1034e−30	2.4684e−28	1.3349e−29	1.2584e−32	
p-value based ANOVA	1.8493e−29	4.7061e−72	4.1940e−43	2.7053e−48	2.9829e−64	
p-value based Kruskal	2.6056e−41	7.2409e−35	2.5984e−32	1.3677e−33	1.3120e−37	

### Application of MWSO for OPF problem

In this subsection, the performance of the MWSO algorithm is compared to the performance of the original WSO in several real-world scenarios to determine whether the proposed algorithm is more successful at solving the OPF problem.

The following mathematical model can be used to express the OPF problem to be solved by the MWSO:

$${\text{Minimize}}:\text{ F}\left({\text{x}},{\text{u}}\right),$$

$$\text{Subject to}:\text{ h}\left({\text{x}},{\text{u}}\right)\le 0,$$

$${\text{g}}\left({\text{x}},{\text{u}}\right)=0,$$

where ${\text{x}}$ and ${\text{u}}$ denote the dependent (state) variables and the independent (control) variables, respectively. While, ${\text{F}}\left({\text{x}},{\text{u}}\right)$ represent the objective functions of the OPF. The objective functions are constrained by set of equality constraints which are represented by ${\text{g}}\left({\text{x}},{\text{u}}\right)$ and set of inequality constraints which are represented by ${\text{h}}\left({\text{x}},{\text{u}}\right)$, as previously presented in “Objective functions and system constraints” section. The control variables of the IEEE 30 bus system are considered the scheduled power of the thermal generators except the swing generator (at bus 1), the scheduled output power of the two wind plants, the scheduled output power of the solar PV plant, and the voltages of all generator buses, while the control variables of the IEEE 57 bus system are similar to the IEEE 30-bus system in addition to the reactive powers of the shunt compensators and the tap changer steps of branch transformers. The cases from 1 to 8 are conducted on the modified IEEE 30 bus power network, while Cases 9 and 10 are dedicated for solving the OPF problem in the IEEE 57 bus power network. The simulation process for these real –world cases is achieved through using the MATLAB software.

#### Case#1: minimizing the total cost

**Figure 9:**
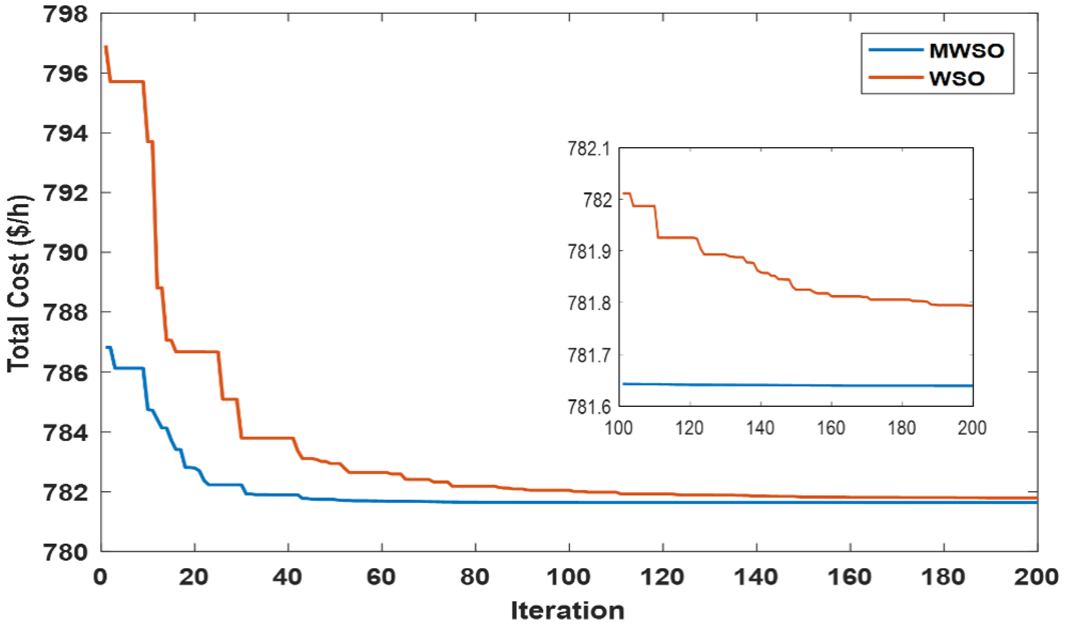
Case 1—solution convergence.

The objective function of this case is to minimize the total production cost from all power sources in the system. The formulation of this objective is based on (22). The values of input parameters required for this case are summarized in Supplementary Material Tables 1A and 2A, while all simulation findings are recorded in Table 5. In comparison to the outcomes of the other techniques utilized, it was discovered through analysis of the findings in Table 5 that the MWSO produced power at the lowest cost, which came to $/h 781.6393. In addition, it was found that the suggested technique has the best convergence for the solution weighed against the WSO, as shown in Fig. 9. Furthermore, looking at the control variables’ limits and the network constraints, all values are within the acceptable limits, as indicated in Table 5.

**Table 5:** Findings of cases#1, 4, 5, and 6.

System parameters	Min.	Max.	Case#1		Case#4		Case#5		Case#6	
			MWSO	WSO	MWSO	WSO	MWSO	WSO	MWSO	WSO
P_Th1_ (MW)	50	140	134.9079	134.9075	123.6238	123.5407	50	50.00038	50.00004	50.09053
P_Th2_ (MW)	20	80	28.68257	28.33868	33.32271	34.19208	46.63944	46.50491	25.51601	29.17295
P_Th3_ (MW)	10	35	10.00003	10.00647	10	10.13565	34.99998	34.99931	34.99994	34.67879
P_schw1_ (MW)	0	75	43.89092	43.54628	46.16204	45.51818	60.2379	67.32408	74.99998	74.90116
P_schw2_ (MW)	0	60	37.03272	36.12722	38.87222	38.89859	45.0599	45.19933	59.99979	59.62789
P_schs_ (MW)	0	50	34.6527	36.2907	36.69981	36.42417	49.57926	42.1811	39.95755	37.05092
V_1_	0.95 (p.u.)	1.1 (p.u.)	1.072697	1.073622	1.070067	1.068522	1.058902	1.060546	1.058482	1.055002
V_2_			1.057523	1.060241	1.056561	1.056407	1.090144	1.090531	1.053107	1.048497
V_5_			1.035992	1.037759	1.035538	1.034382	1.039766	1.040965	1.04382	1.043851
V_8_			1.039669	1.05288	1.09986	1.062879	1.058556	1.041715	1.049941	1.043217
V_11_			1.098002	1.085739	1.098908	1.097121	1.091949	1.008489	1.1	1.095706
V_13_			1.052598	1.031306	1.050376	1.05858	0.956792	1.031062	1.058634	1.095327
Q_Th1_ (MVAr)	− 20	150	− 1.24438	− 3.82612	− 3.04847	− 6.55793	− 20	− 20	− 5.11021	− 3.02559
Q_Th2_ (MVAr)	− 20	60	12.80724	20.18123	10.90574	13.3021	60	60	7.182402	0.861426
Q_Th3_ (MVAr)	− 15	40	34.97329	40	40	40	40	36.57897	37.90647	31.06411
Q_schw1_ (MVAr)	− 30	35	23.78768	24.20308	22.23821	21.231	16.55817	15.12577	20.86855	25.65432
Q_schw2_ (MVAr)	− 25	30	30	26.78253	30	29.1542	30	3.007821	30	30
Q_schs_ (MVAr)	− 20	25	16.65224	9.573353	15.5807	18.5711	− 13.216	13.83011	18.44549	25
Total power cost ($/h)			781.6393	781.7939	810.3348	810.6727	866.3527	867.3017	881.2034	879.3627
Emissions (tonne/h)			1.762039	1.762073	0.8964	0.8919	0.0958327	0.095833	0.098817	0.09794
Emission tax ($/tonne)			0	0	20	20	0	0	0	0
Emissions cost ($/h)			0	0	17.9281	17.8388	0	0	0	0
P_loss_ (MW)			5.766852	5.816821	5.280536	5.3093	3.114166	2.815226	2.073312	2.122236
V_d_ (p.u.)			0.458416	0.437043	0.4601	0.4809	0.730868	0.55025	0.513365	0.526667

#### Case#2: changing the value of reserve cost coefficient

**Figure 10:**
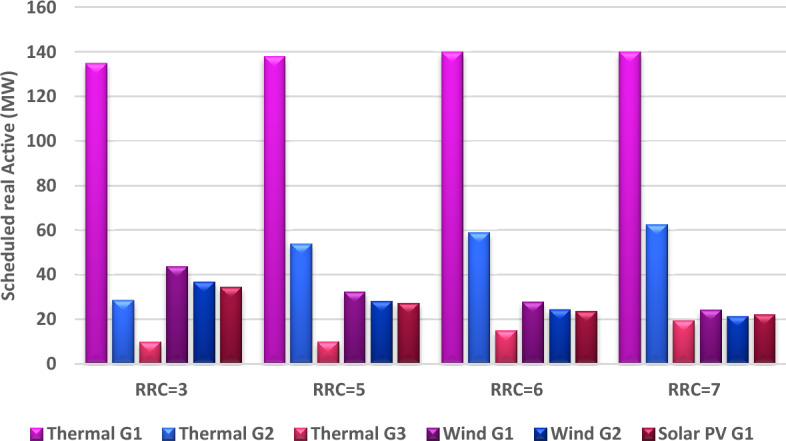
Optimal scheduled active powers of all generators with different reserve cost coefficient.

**Figure 11:**
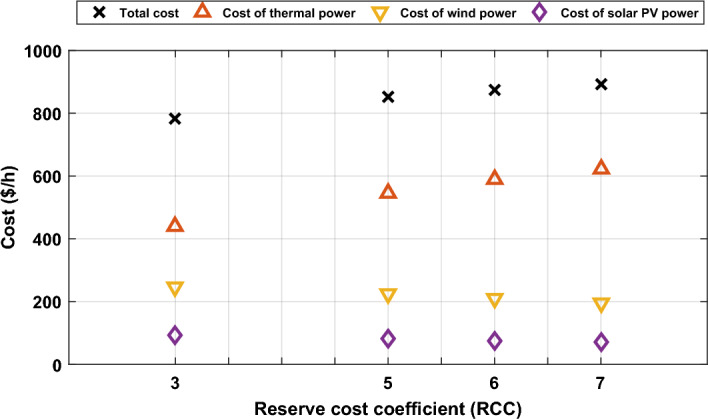
Impact of various reserve cost coefficients (RCC) on various costs.

In Case #1, the reserve cost coefficient for the two wind plants and the solar plant was constant value equals to 3 for all of them. In this case, the value of this coefficient will be changed from 3 to 5, 6, and 7 to study the effect of this change on the optimal cost of production. The value of penalty charge coefficient for both wind and solar plants is constant in this case at 1.5. The optimal schedule of output power of all generators is determined at each value of the reserve cost coefficient as a subcase. This optimal schedule is highlighted by Fig. 10. As anticipated, an escalation in the reserve charge coefficient led to a drop in the planned output of wind and solar power facilities. This drop can be explained as decreasing the schedule of renewable power will decrease the reserve charge in the event of overestimation. In contrast, the schedule of thermal power will increase due to reducing the schedule of renewable power. Consequently, the cost of production from renewable energy will decrease, while the cost of production from thermal generators will increase and the total cost of production from all generators will increase as indicated in Fig. 11.

#### Case#3: changing the value of penalty cost coefficient

**Figure 12:**
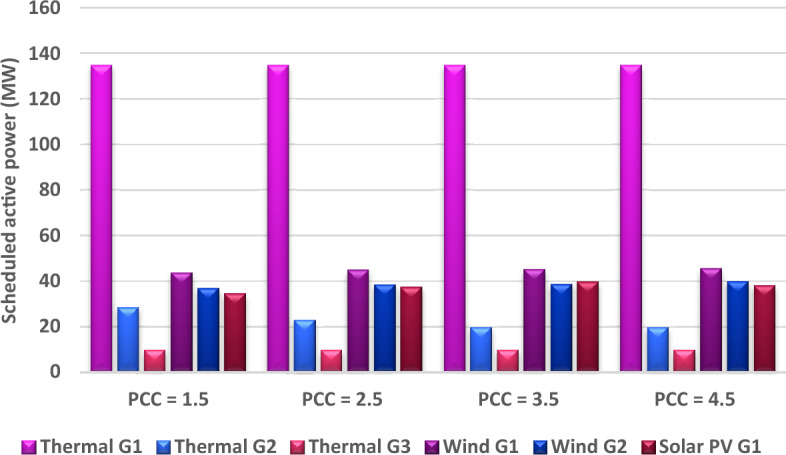
Optimal scheduled active powers of all generators with different penalty cost coefficient.

**Figure 13:**
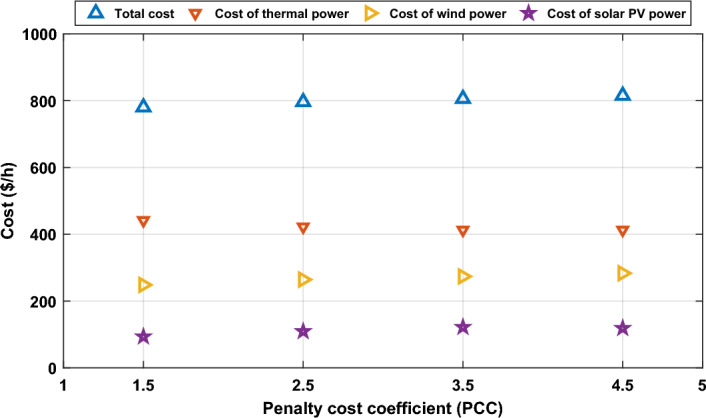
Impact of various penalty cost coefficients (PCC) on various costs.

This case is similar to Case 2, the only difference is varying the penalty cost coefficient with maintain the reserve cost coefficient constant to study the influence of changing the penalty charge coefficient on the schedule of power in the system and the associated costs of production. The values of the penalty cost coefficient are changed from 1.5 in Case 1 to be 2.5, 3.5, and 4.5, respectively. Each value from these values is considered as a subcase, and the results of schedule power and production cost is obtained. To analyse these results, the schedule powers from all generators are illustrated in Fig. 12. As anticipated, with escalating the penalty charge coefficient, the schedule power from renewable energy resources increases to minimize the penalty fees in the event of underestimation of renewable power. This increase in the schedule of renewable energy resulted in reducing the schedule power from thermal plants. This change in the scheduled powers will consequently be translated into the cost of production as shown in Fig. 13, where the cost of wind and solar power increases, while the cost of thermal power decreases, but the total cost of production will increase.

#### Case#4: impact of forcing carbon tax

**Figure 14:**
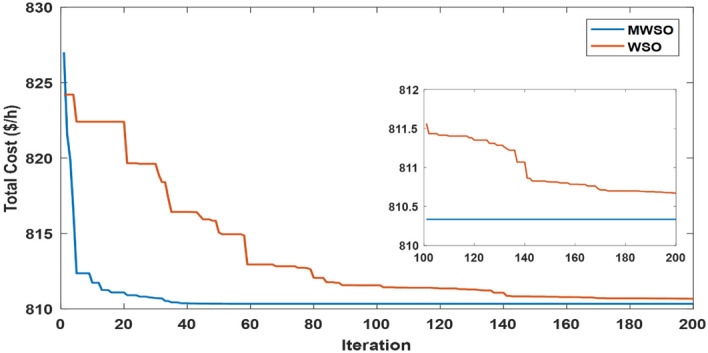
Case 4-solution convergence.

The MWSO is applied to examine the impact of placing a tax on emissions from thermal energy generation in this scenario. The objective function is minimizing the total production cost with the existing carbon tax based on (23). All input parameters are set to the same values as in Supplementary Materials Tables 1A and 2A, except for the carbon tax, which is set at $20 per tonne. The purpose of imposing a tax on emissions is to reduce energy production from thermal sources and increase reliance on renewable energy sources. To ensure that the imposition of this tax achieved its goal, the results of this case, which are listed in Table 5, were examined, and it was observed that production from thermal energy sources was actually reduced while production from renewable energy sources increased compared to the first case in which no tax was imposed. As in the previous case study, the MWSO achieved the lowest production cost ($/h 810.3348) with the fastest solution convergence, as shown in Fig. 14 as well as all values for constraints inside the acceptable range.

#### Case#5: minimizing carbon emissions

**Figure 15:**
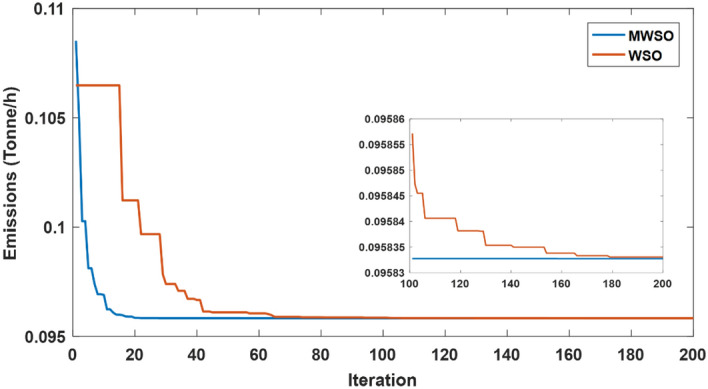
Case#5-solution convergence.

This case study was assigned to employ the suggested strategy (MWSO) according to Eq. (24) to lessen emissions since the system under investigation uses three thermal energy sources that emit a significant amount of greenhouse gases. In this situation, lowering emissions is the main objective, regardless of the cost of production. Therefore, it is evident from Table 5 that the emissions are minimized, while the total cost increases compared to Case#1. It is also noted that MWSO has outperformed the original WSO in minimizing the carbon emissions and convergence characteristics as illustrated in Fig. 15.

#### Case#6: minimizing power losses

**Figure 16:**
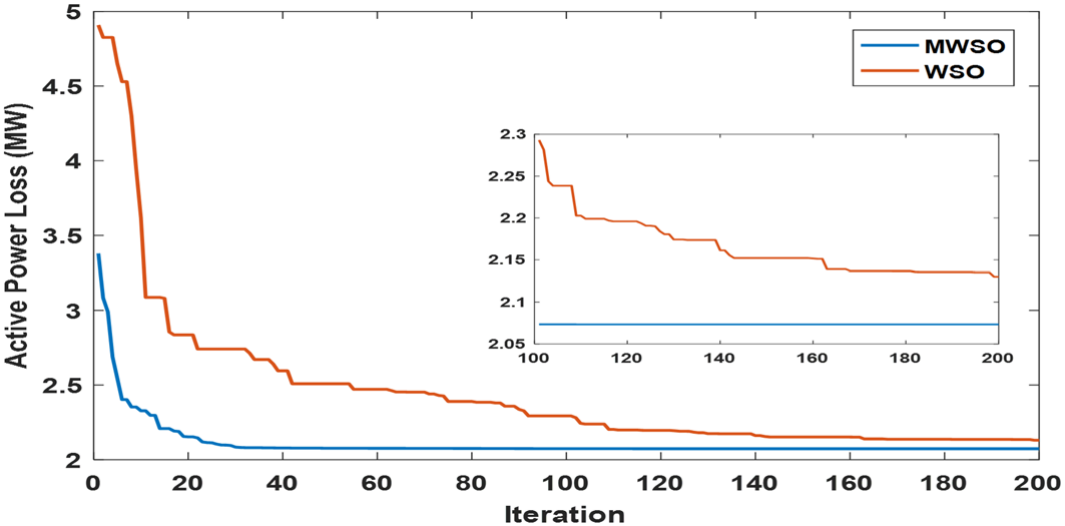
Case#6-Solution Convergence.

**Figure 17:**
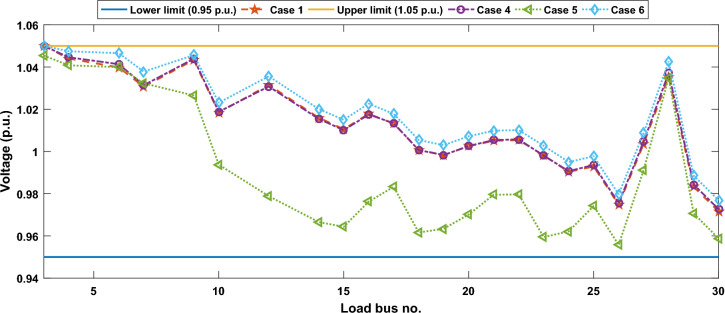
Voltage profile of load buses—Cases#1, 4, 5, and 6.

Another important objective of OPF is minimizing the active power losses. This objective is performed in this case study according to (26). The obtained result of this case is indicated in Table 5 and Fig. 16. It is observed from these outcomes that the minimum power loss is achieved by the MWSO with fast convergence compared to the WSO. The voltage profile of load buses voltage for the Cases 1, 4, 5, and 6 is indicated by Fig. 17. The voltage profile shows that all voltages of load buses are within the allowed values.

#### Case#7: ramp rate of thermal generators

**Figure 18:**
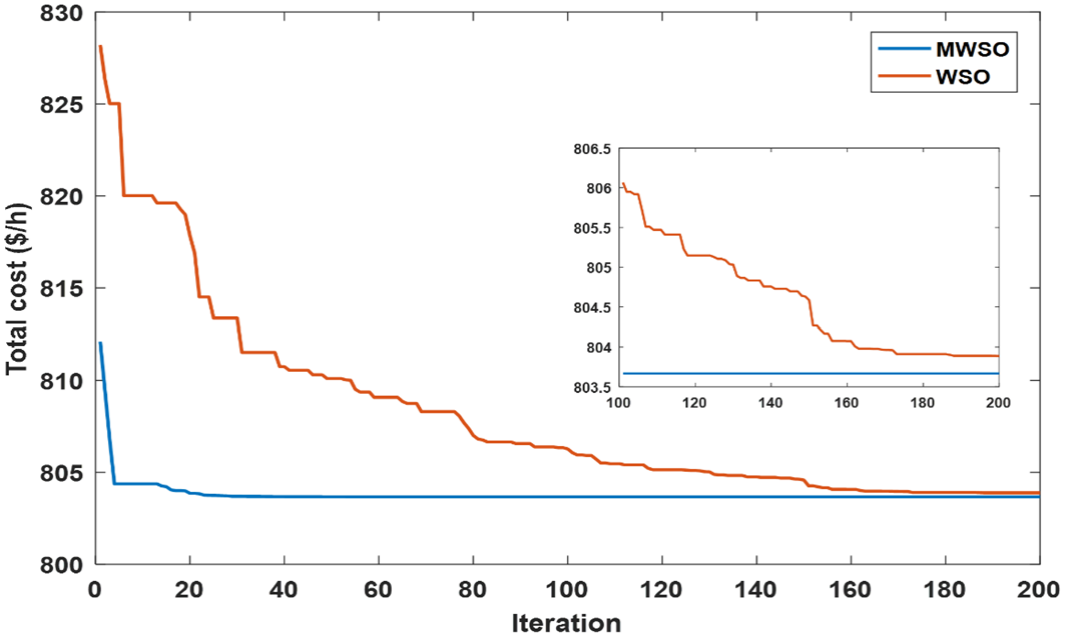
Case#7—solution convergence.

**Figure 19:**
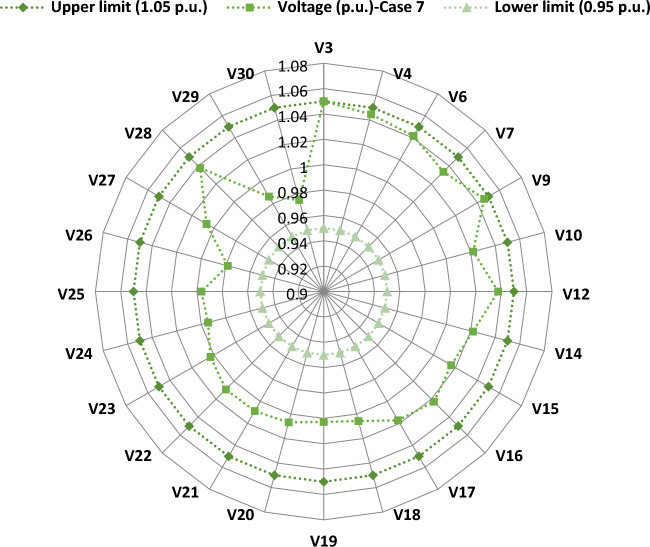
Voltage profile of load buses—Case#7.

The limits of ramp rate for thermal generators can change the optimal solution of optimal power flow problem, thus this case study is dedicated to study their impact on the OPF problem. The input factors of this case are the same as in Supplementary Material Table 1A, while the output power at the preceding hour and the ramp rate limits for each thermal generator are indicated in Supplementary Material Table 2A. The simulation findings for this situation are indicated in Table 6. MWSO has achieved the lowest cost of production when compared to WSO, and it is faster to converge, as indicated in Fig. 18. Most notably, the total production cost increased from 781.6393 in Case 1 to 803.6681 in this case, as was to be expected, as the limits of operation of the thermal generators were changed in this case. Additionally, the voltage profile of load buses voltage for Case 7 is indicated by Fig. 19. It demonstrates that every voltage of the load buses is within the allowed range.

**Table 6:** Case#7’s findings.

Control variables and parameters	Min.	Max.	MWSO	WSO
P_Th1_ (MW)	79.211	114.211	94.4917	95.53566
P_Th2_ (MW)	65	80	65	65.00082
P_Th3_ (MW)	12	24	12	12.02091
P_schw1_ (MW)	0	75	44.02366	42.28944
P_schw2_ (MW)	0	60	37.21468	38.01709
P_schs_ (MW)	0	50	35.46039	35.42991
V_1_ (p.u.)	0.95	1.1	1.069842	1.065352
V_2_ (p.u.)	0.95	1.1	0.994809	0.987169
V_5_ (p.u.)	0.95	1.1	1.040063	1.056223
V_8_ (p.u.)	0.95	1.1	1.09463	1.062794
V_11_ (p.u.)	0.95	1.1	1.1	1.098422
V_13_ (p.u.)	0.95	1.1	1.062945	1.094922
Q_Th1_ (MVAr)	− 20	150	13.9765	6.048195
Q_Th2_ (MVAr)	− 20	60	− 20	− 20
Q_Th3_ (MVAr)	− 15	40	40	35
Q_schw1_ (MVAr)	− 30	35	30.17699	40
Q_schw2_ (MVAr)	− 25	30	29.74575	28.39266
Q_schs_ (MVAr)	− 20	25	20.07812	25
Total power cost ($/h)			803.6681	803.8867
Emissions (tonne/h)			0.221603	0.230155
P_loss_ (MW)			4.790434	4.893833
V_d_ (p.u.)			0.503734	0.551338

#### Case#8: uncertainty of load demand

**Figure 20:**
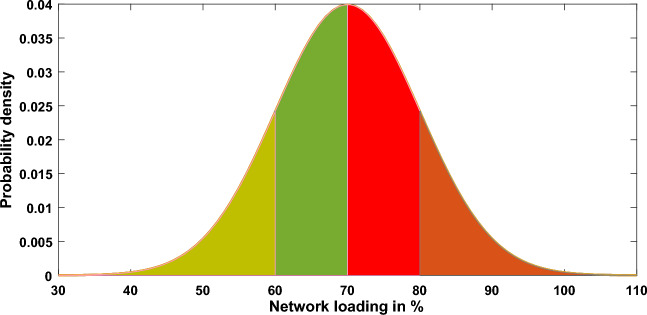
Normal PDF of network loading.

Another important factor that may influence the solution of the optimal power flow problem is the uncertainty in the load demand, so this case study was dedicated to figuring out the OPF problem using the proposed method (MWSO) in this situation. This helps assess how well the proposed method works for figuring out the OPF problem in some complex scenarios that include changes in both the source and the load. For modelling the uncertainty of load demand, a normal PDF is used^61^ as shown in Fig. 20. The selected values of the standard deviation $({\upsigma }_{{\text{ld}}})$ and the mean $({\upmu }_{{\text{ld}}})$ for the normal PDF are 10 and 70, respectively. Each loading level (scenario) has a probability of occurrence, this probability (${\Delta }_{{\text{ld}},{\text{i}}}$) can be calculated as follows:

60 $${\Delta }_{ld,i}=\int \limits_{{P}_{ld,i}^{low}}^{{P}_{ld,i}^{high}}\frac{1}{{\sigma }_{ld}\surd 2\pi }exp\left[\frac{{\left({P}_{ld}-{\mu }_{ld}\right)}^{2}}{{2{\sigma }_{ld}}^{2}}\right]d{P}_{ld} .$$

Here, ${P}_{ld}$ denotes the system loading, while ${P}_{ld,i}^{high}$ and ${P}_{ld,i}^{low}$ denotes the upper and lower limits of the loading level. While the mean of occurring a certain loading level (${P}_{ld,i}^{-}$) can be determined as follows:

61 $${P}_{ld,i}^{-}=\frac{1}{{\Delta }_{ld,i}}\int \limits_{{P}_{ld,i}^{low}}^{{P}_{ld,i}^{high}}\left(\frac{1}{{\sigma }_{ld}\surd 2\pi }exp\left[\frac{{\left({P}_{ld}-{\mu }_{ld}\right)}^{2}}{{2{\sigma }_{ld}}^{2}}\right]\right)d{P}_{ld }.$$

**Figure 21:**
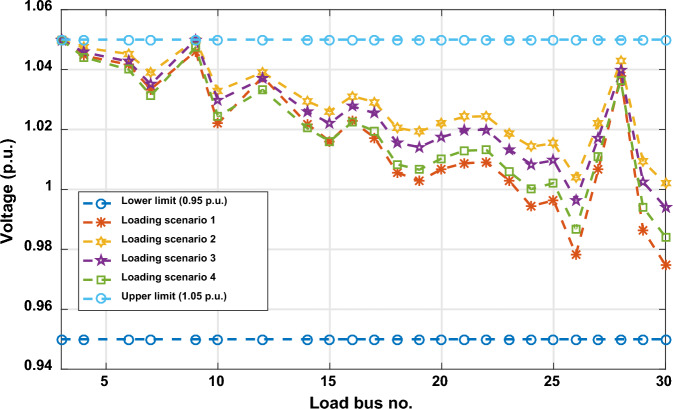
Voltage profile of load buses—Case#8.

The estimated means (in percentages of nominal system loading, ${{\text{P}}}_{{\text{ld}}}$) and the likelihoods for the four loading scenarios are indicated in Table 7. The outcomes of solving the OPF in this case using the WSO and the MWSO are listed in Table 8. The outcomes demonstrate once more how much more successful the MWSO is in this more complicated case when compared to the conventional WSO. For this case, the voltage profile of load buses through the four different loading scenarios is indicated by Fig. 21. It demonstrates that every voltage of the load buses is within the allowed range.

**Table 7:** Means and probabilities of different loading scenarios.

Loading scenario $(i)$	%Loading, ${P}_{ld,i}^{-}$(Mean)	Probability,${\Delta }_{ld,i}$
1	54.749	0.15866
2	65.401	0.34134
3	74.599	0.34134
4	85.251	0.15866

**Table 8:** Findings of Case#8.

Control variables and parameters	Min.	Max.	Loading scenario 1		Loading scenario 2		Loading scenario 3		Loading scenario 4	
			MWSO	WSO	MWSO	WSO	MWSO	WSO	MWSO	WSO
P_Th1_ (MW)	50	140	50	50.01655	54.07997	51.67429	93.7968	95.3039	134.9079	134.5669
P_Th2_ (MW)	20	80	20	20.00845	21.26297	20.82011	20	20.06234	20	20.00968
P_Th3_ (MW)	10	35	10.00003	10.00353	10	10.0105	10	10.00681	10	10.01678
P_schw1_ (MW)	0	75	27.90323	28.05193	38.18699	8.34834	32.59863	31.58711	29.07629	30.34143
P_schw2_ (MW)	0	60	24.13581	24.14331	32.82259	32.52077	27.99681	27.59591	24.90125	25.90116
P_schs_ (MW)	0	50	24.28961	24.10909	30.42127	33.37306	29.95587	29.95407	27.91059	25.90832
V_1_	0.95 (p.u.)	1.1 (p.u.)	1.058372	1.054062	1.05789	1.056401	1.064087	1.055435	1.071502	1.074762
V_2_			0.95	1.050876	1.051742	1.050438	1.053308	1.043473	1.056042	1.056641
V_5_			1.042923	1.041658	1.041144	1.040075	1.037112	1.030677	1.034114	1.03289
V_8_			1.045115	1.046493	1.044227	1.043993	1.041447	1.027535	1.038842	1.038537
V_11_			1.08286	1.030163	1.089735	1.083579	1.097709	1.068359	1.1	1.089002
V_13_			1.051122	1.045835	1.050416	1.06136	1.050282	1.051831	1.049016	1.037021
Q_Th1_ (MVAr)	− 20	150	5.884286	− 10.4512	− 5.25441	− 5.73143	− 4.05866	− 1.30126	− 1.59105	5.705728
Q_Th2_ (MVAr)	− 20	60	− 20	4.489927	0.595184	− 0.59709	4.26284	2.746339	10.15932	7.98974
Q_Th3_ (MVAr)	− 15	40	15.07408	24.48701	17.81097	17.89279	20.90301	16.28518	25.66326	28.10263
Q_schw1_ (MVAr)	− 30	35	13.17092	10.67888	11.62395	11.32741	14.82334	20.15231	18.97342	17.52495
Q_schw2_ (MVAr)	− 25	30	17.64633	1.202837	21.79776	19.26104	25.8862	19.12294	28.41637	25.46312
Q_schs_ (MVAr)	− 20	25	7.794191	8.896721	9.111757	13.45866	10.57623	16.19786	12.32565	8.875978
Total power cost ($/h)			409.4783	410.1268	495.5356	495.8449	575.9702	576.2665	652.2	652.4366
Emission (tonne/h)			0.104028	0.104032	0.105843	0.104582	0.218928	0.230906	1.764577	1.727937
P_loss_ (MW)			1.171063	1.175182	1.426278	1.399564	2.935609	3.097638	5.193878	5.142087
V_d_ (p.u.)			0.751873	0.546953	0.658447	0.687879	0.578764	0.387242	0.491005	0.41651

#### Case#9: minimization of total production cost in IEEE 57-bus system

This case study was created to determine the validity of the MWSO in tackling the problem of OPF in the most complex systems by minimizing the cost of power production in the standard IEEE-57 system. Based on (22), the objective function is the same as in the IEEE-30 bus system. The system restrictions are identical to those of the IEEE-30 bus system. The IEEE-57 bus system has been upgraded to include four thermal generators linked at buses 1 (swing), 3, 8, and 12, two wind plants linked at buses 2 and 6, and a solar PV plant linked at bus 9. The cost and emission coefficients of thermal generators in this system are detailed in Supplementary Material Table 3A, while the parameters of Weibull and lognormal PDF are provided in Supplementary Material Table 4A. The load of this system is 1250.8 MW for active power and 336.4 MVA for reactive power. The simulation findings of this case are listed in Table 9.

**Table 9:** Findings of Case#9.

	Min.	Max.	Case#9						
			MWSO	WSO		Min.	Max.	MWSO	WSO
Control variables					Control variables				
P_Th2_ (MW)	40	140	116.3108	118.8395	T_37_ (p.u.)	0.9	1.1	1.0131	0.9632
P_Th3_ (MW)	100	550	335.1005	334.3525	T_41_ (p.u.)			0.9951	0.9675
P_Th4_ (MW)	100	410	409.9838	409.2727	T_46_ (p.u.)			0.9555	0.9685
P_schw1_ (MW)	30	100	99.99995	99.99619	T_54_ (p.u.)			0.9121	1.0119
P_schw2_ (MW)	30	100	100	99.95285	T_58_ (p.u.)			0.9855	0.9994
P_schs_ (MW)	30	100	100	99.9921	T_59_ (p.u.)			0.9687	0.9754
V_1_ (p.u.)	0.95	1.1	1.070023	1.018097	T_65_ (p.u.)			0.9745	1.0188
V_2_ (p.u.)			1.069468	1.021857	T_66_ (p.u.)			0.9408	0.9243
V_3_ (p.u.)			1.065383	1.034479	T_71_ (p.u.)			0.9740	0.9480
V_6_ (p.u.)			1.06352	1.047952	T_73_ (p.u.)			0.9947	0.9431
V_8_ (p.u.)			1.067031	1.046085	T_76_ (p.u.)			0.9594	1.0081
V_9_ (p.u.)			1.047145	1.025675	T_80_ (p.u.)			0.9866	1.0450
V_12_ (p.u.)			1.052097	1.03748	Parameters				
$${Q}_{C18}$$(MVAr)	0	20	3.6528	11.4460	P_Th1_ (MW)	0	576	100.0003	100.8469
$${Q}_{C25}$$(MVAr)			13.9093	12.8466	Q_Th1_ (MVAr)	− 140	200	51.0892	− 10.5250
$${Q}_{C53}$$(MVAr)			12.4491	7.0037	Q_Th2_ (MVAr)	− 10	60	34.6411	39.8221
T_19_ (p.u.)	0.9	1.1	1.0734	0.9797	Q_Th3_ (MVAr)	− 140	200	39.8175	41.7818
T_20_ (p.u.)			0.9214	1.0708	Q_Th4_ (MVAr)	− 150	155	42.9346	95.7378
T_31_(p.u.)			1.0119	1.0039	Q_schw1_ (MVAr)	− 17	50	49.9987	46.7703
T_35_ (p.u.)			1.0411	0.9294	Q_schw2_ (MVAr)	− 8	25	− 1.7200	21.1087
T_36_ (p.u.)			0.9815	0.9538	Q_schs_ (MVAr)	− 3	9	8.9967	3.9104
Total production cost ($/h)			20,229.82	20,269.83					
Emissions (tonne/h)			0.983993	0.982647					
P_loss_ (MW)			10.59528	12.45273					
V_d_ (p.u.)			1.76683	1.192722					

**Figure 22:**
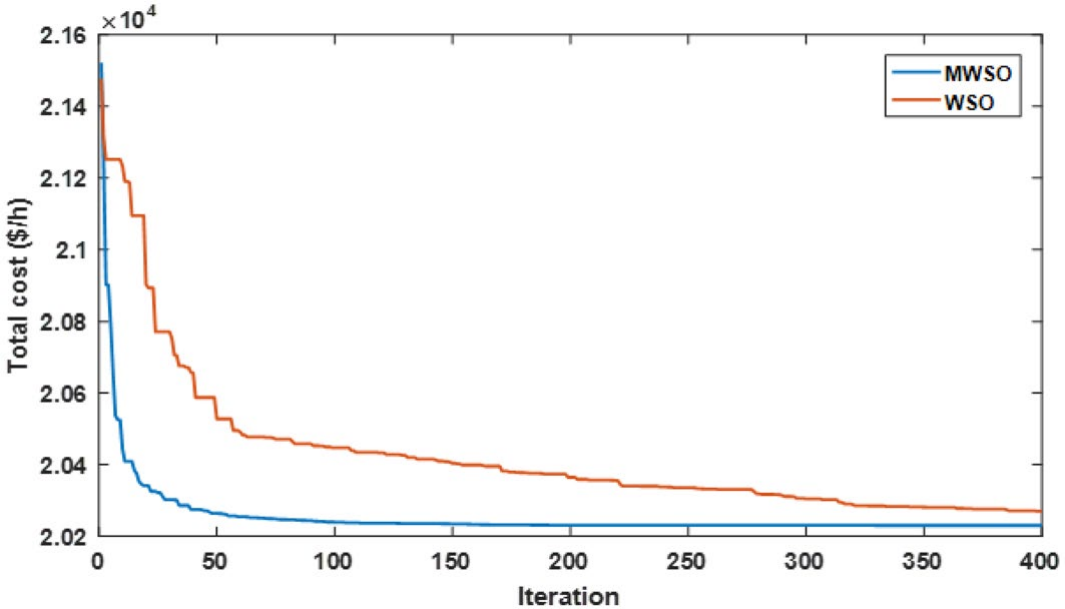
Case#9—solution convergence.

**Figure 23:**
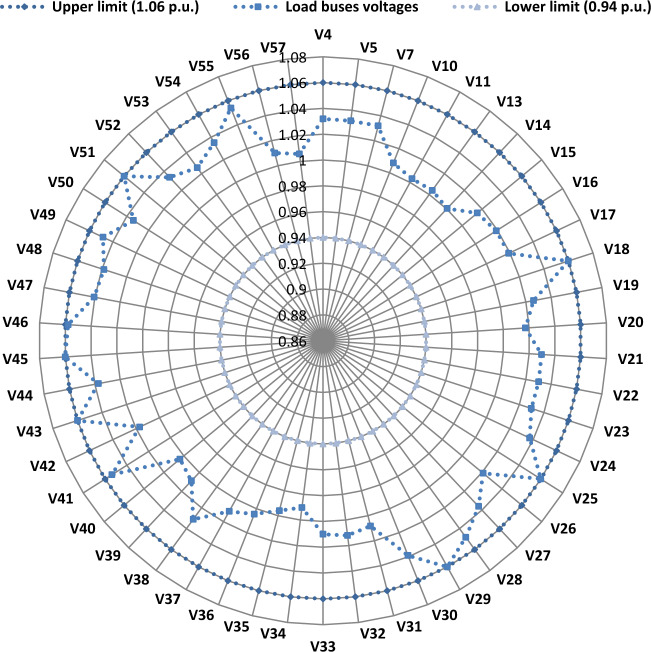
Voltage profile of load buses—Case#9.

The findings of this complicated case clearly prove the success of the MWSO in minimizing the total cost of production with high convergence characteristics, as shown in Fig. 22, compared to the original WSO. The voltage profile of load buses of the IEEE 57 bus network is indicated by Fig. 23. It shows that all load buses voltages are within the allowed values.

#### Case#10: minimization of total production cost with carbon tax in IEEE 57-bus system

**Figure 24:**
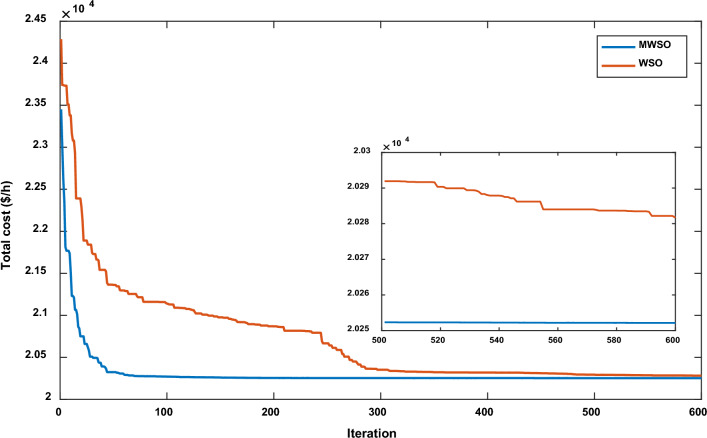
Case#10—solution convergence.

**Figure 25:**
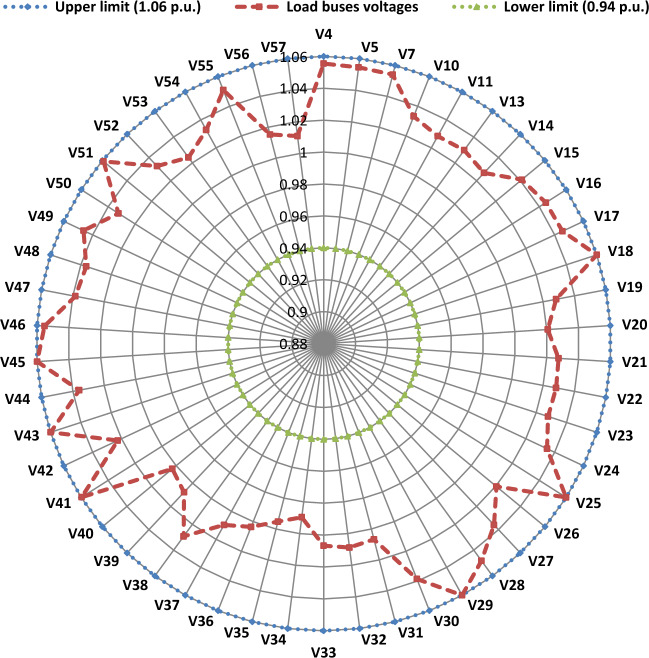
Voltage profile of load buses—Case#10.

In this case, more complicated objective function is defined for minimizing the total cost with enforcing a tax on carbon emissions of thermal generator in the IEEE 57-bus system. The formulation of the objective function is the same as in (23). The parameters of this case are the same as in Case #9, and the carbon tax is set at $20 per tonne. This case is performed for 10 runs with 600 iterations for each run. The findings of this case are presented in Table 10. The convergence curves of the MWSO and WSO for this case are illustrated by Fig. 24. It also proofs that the MWSO has minimized the total cost with fast convergence compared to the original WSO. The voltages of the load buses are also within the allowed limits as shown by Fig. 25.

**Table 10:** Findings of Case#10.

	Min.	Max.	Case#10						
			MWSO	WSO		Min	Max	MWSO	WSO
Control variables					Control variables				
P_Th2_ (MW)	40	140	116.6172	124.1748	T_37_ (p.u.)	0.9	1.1	1.0126	1.0336
P_Th3_ (MW)	100	550	334.8935	328.5204	T_41_ (p.u.)			0.9907	1.0006
P_Th4_ (MW)	100	410	409.9421	409.8027	T_46_ (p.u.)			0.9618	0.9609
P_schw1_ (MW)	30	100	100	99.9861	T_54_ (p.u.)			0.9091	0.9225
P_schw2_ (MW)	30	100	100	99.9534	T_58_ (p.u.)			0.9779	0.9503
P_schs_ (MW)	30	100	100	99.9522	T_59_ (p.u.)			0.9646	0.9588
V_1_ (p.u.)	0.95	1.1	1.0593	1.0314	T65 (p.u.)			0.9699	0.9668
V_2_ (p.u.)			1.0596	1.0259	T66 (p.u.)			0.9373	0.9517
V_3_ (p.u.)			1.0584	1.0211	T71 (p.u.)			0.9683	0.9719
V_6_ (p.u.)			1.0589	1.0385	T73 (p.u.)			0.9897	1.0294
V_8_ (p.u.)			1.0631	1.0556	T76 (p.u.)			0.9581	0.9370
V_9_ (p.u.)			1.0423	1.0318	T80 (p.u.)			0.9879	1.0364
V_12_ (p.u.)			1.0463	1.0380	Parameters				
$${Q}_{C18}$$(MVAr)	0	20	7.6015	13.8825	P_Th1_ (MW)	0	576	99.9993	100.1232
$${Q}_{C25}$$(MVAr)			15.3195	10.9314	Q_Th1_ (MVAr)	− 140	200	40.5944	49.3105
$${Q}_{C53}$$(MVAr)			12.7069	14.1442	Q_Th2_ (MVAr)	− 10	60	32.4493	− 9.4883
T_19_ (p.u.)	0.9	1.1	0.9559	1.0785	Q_Th3_ (MVAr)	− 140	200	42.9723	68.9071
T_20_ (p.u.)			1.0144	0.9719	Q_Th4_ (MVAr)	− 150	155	47.2361	78.3332
T_31_(p.u.)			1.0023	1.0449	Q_schw1_ (MVAr)	− 17	50	49.9212	33.7027
T_35_ (p.u.)			0.9493	1.0099	Q_schw2_ (MVAr)	− 8	25	− 2.4103	− 1.341
T_36_ (p.u.)			1.1	0.9664	Q_schs_ (MVAr)	− 3	9	8.9899	7.7592
Total production cost ($/h)			20,252.15028	20,281.7073					
Emissions (tonne/h)			0.98356	0.97234					
P_loss_ (MW)			10.6519	11.7129					
V_d_ (p.u.)			1.6673	0.99334

### Statistical analysis

In this section, a statistical summary is presented in Table 11 for the case studies from 1 to 10 among the implemented simulation runs for each case. In addition to that, Wilcoxon signed rank test is carried out to compare between the MWSO and WSO as presented in Table 12. The column ${H}_{O}$ in this table specifies whether or not the null hypothesis is correct. The effectiveness of the two algorithms is statistically similar for the study instance if the null hypothesis is true (i.e., ${H}_{O}$ = Yes, with a threshold of significance = 0.05).

**Table 11:** Statistical analysis.

Case	Sub-case	No. of runs	MWSO				WSO			
			Best	Worst	Mean	Std	Best	Worst	Mean	Std
Case 1		25	781.6393	784.337	782.5262	0.53929	781.7939	787.3396	783.4849	1.443481
Case 4			810.3348	811.6798	811.1019	0.264085	810.6727	814.2481	811.7952	0.79011
Case 5			0.095833	0.095833	0.095833	0	0.095833	0.098451	0.095998	0.000508
Case 6			2.073312	2.159843	2.107706	0.023982	2.122236	2.573161	2.2464	0.09928
Case 7			803.6681	805.3186	804.7313	0.428817	803.8867	806.8586	805.3376	0.683246
Case 8	LS 1		409.4783	411.2542	410.1465	0.373602	410.1268	411.7695	410.7595	0.469976
	LS 2		495.5356	497.2558	496.2514	0.387962	495.8449	498.361	496.6008	0.528525
	LS 3		575.9702	577.6532	576.7206	0.334927	576.2665	578.5848	577.3095	0.504536
	LS 4		652.2	654.8222	653.0348	0.605697	652.4366	655.2544	653.726	0.686525
Case 9		10	20,229.82477	20,252.326	20,236.0143	7.74938	20,269.8	20,393.4	20,312.4	42.28056
Case 10			20,252.1503	20,263.004	20,255.2116	2.8515	20,281.7073	20,332.0584	20,311.1423	15.265

**Table 12:** Results of Wilcoxon signed rank test.

Case	Sub-case	MWSO versus WSO			
		R+	R−	p-value	H_0_
Case 1		273	3	0.00004	No
Case 4		234	42	0.00350	No
Case 6		270	6	0.00006	No
Case 7		276	0	0.00003	No
Case 8	LS 1	258	18	0.00026	No
	LS 2	276	0	0.00003	No
	LS 3	262	14	0.00016	No
	LS 4	276	0	0.00003	No
Case 9		55	0	0.00195	No
Case 10		55	0	0.00195	No

$R+$ is the sum of the rankings for runs in which MWSO exceeds WSO, while $R-$ denotes the rankings for runs in which WSO exceeds MWSO. The p-value establishes the importance of results. The lower the p-value, the stronger the argument against the null hypothesis (${H}_{O}$). The results show that MWSO outperformed WSO in all cases, as the p-value is lower than 0.05 and there are no null hypotheses.

## Conclusion

This paper has introduced a modified white shark optimization (WSO) algorithm for optimizing power flow problems. The modified algorithm incorporates Gaussian barebones and quasi-oppositional learning mechanisms to improve its performance. The MWSO algorithm is tested using the CEC2017 benchmark functions and compared against six other efficient algorithms. The results show superior performance, making it well-suited for addressing power flow optimization problems, especially in renewable energy sources with intermittent output and fluctuating load demands. The paper introduces probabilistic models for solar and wind power using Weibull and lognormal PDFs, and presents a normal PDF-based probabilistic model for load demand. The MWSO algorithm is applied to solve the power flow optimization problem in two modified IEEE standard test systems. In the IEEE 30-bus system, it is used to minimize the total generation cost, both with and without considering carbon tax on emissions, while simultaneously minimizing active power losses. The study investigates the impact of varying reserve and penalty costs for overestimating and underestimating wind and solar power output, four different load scenarios, and the influence of imposing ramp rate limits of thermal generators on the optimal power flow problem. To validate the robustness of the proposed algorithm in more complex systems, the IEEE 57-bus network is also modified and subjected to the MWSO algorithm for solving the power flow optimization problem. The simulation results, statistical analysis, and the Wilcoxon signed rank test confirm the superiority and effectiveness of the MWSO algorithm in addressing power flow optimization problems.

### Supplementary Information

Supplementary Tables.
