# Supplementary material for: A modified white shark optimizer for optimal power flow considering uncertainty of renewable energy sources
Source: Sci Rep. 2024 Feb 6;14:3051. doi: 10.1038/s41598-024-53249-z (PMC10847516; doi:10.1038/s41598-024-53249-z)
Supplement: Supplementary file 1 — Supplementary Tables. [file 41598_2024_53249_MOESM1_ESM.docx]

Table 1A. Wind and solar plants data-IEEE-30 bus system.

| *Wind plants* | | | | |
| --- | --- | --- | --- | --- |
| Wind plant # | No. of wind Turbines | Rated power | Weibull Parameters | Weibull mean, $M_{weibull}$ |
| 1 | 25 | 75 MW | k=2, c=9 | $\mathrm{Wd}_{v}$= 7.976 (m/s) |
| 2 | 20 | 60 MW | k=2, c=10 | $\mathrm{Wd}_{v}$= 8.862 (m/s) |
| *Solar PV plants* | | | | |
| Solar plant # | Rated power | | Lognormal parameters | Lognormal mean, $M_{lgn}$ |
| 1 | 50 MW | | δ=0.6, µ=6 | I=483 (W/m^2^) |

Table 2A. Parameters of thermal plants-IEEE 30 bus power system.

| ${Th}_{i}$ | Bus | $d_{Th}$ | $e_{Th}$ | $a_{Th}$ | $b_{Th}$ | $c_{Th}$ | $\emptyset_{Th}$ | $\gamma_{Th}$ | $\psi_{Th}$ | $\zeta_{Th}$ | $\tau_{Th}$ | $P_{Thi}^{0}$ (MW) | ${Ur}_{i}$ (MW) | ${Dr}_{i}$ (MW) |
| --- | --- | --- | --- | --- | --- | --- | --- | --- | --- | --- | --- | --- | --- | --- |
| 1 | 1 | 18 | 0.037 | 30 | 2 | 0.00375 | 4.091 | 6.49 | −5.554 | 6.667 | 0.0002 | 99.211 | 15 | 20 |
| 2 | 2 | 16 | 0.038 | 25 | 1.75 | 0.0175 | 2.543 | 5.638 | −6.047 | 3.333 | 0.0005 | 80 | 10 | 15 |
| 3 | 8 | 12 | 0.045 | 20 | 3.25 | 0.00834 | 5.326 | 3.38 | −3.55 | 2 | 0.002 | 20 | 4 | 8 |

Table 3A. Cost coefficients of thermal plants for the IEEE 57 bus network.

| ${Th}_{i}$ | Bus | $d_{Th}$ | $e_{Th}$ | $a_{Th}$ | $b_{Th}$ | $c_{Th}$ | $\emptyset_{Th}$ | $\gamma_{Th}$ | $\psi_{Th}$ | $\zeta_{Th}$ | $\tau_{Th}$ |
| --- | --- | --- | --- | --- | --- | --- | --- | --- | --- | --- | --- |
| 1 | 1 | 18 | 0.037 | 0 | 20 | 0.0775795 | 4.091 | 6.49 | −5.554 | 2.857 | 0.0002 |
| 2 | 3 | 13.5 | 0.041 | 0 | 20 | 0.25 | 6.131 | 5.151 | 5.555 | 6.667 | 0.00001 |
| 3 | 8 | 14 | 0.04 | 0 | 20 | 0.0222222 | 4.258 | 4.586 | 5.094 | 8 | 0.000001 |
| 4 | 12 | 12 | 0.045 | 0 | 20 | 0.0322581 | 5.326 | 3.38 | 3.555 | 2 | 0.002 |

Table 4A. Wind and solar plants data- IEEE -57 bus system.

| Wind plants | | | | |
| --- | --- | --- | --- | --- |
| Wind plant # | No. of wind Turbines | Rated power | Weibull Parameters | Weibull mean, $M_{weibull}$ |
| 1 | 25 | 100 MW | k=2, c=9 | $\mathrm{Wd}_{v}$ = 7.976 (m/s) |
| 2 | 20 | 100 MW | k=2, c=10 | $\mathrm{Wd}_{v}$= 8.862 (m/s) |
| Solar PV plants | | | | |
| Solar plant # | Rated power | | Lognormal parameters | Lognormal mean, $M_{lgn}$ |
| 1 | 100 MW | | δ=0.6, µ=6 | I=483 (W/m^2^) |
